# Muscle-strengthening activities are associated with lower risk and mortality in major non-communicable diseases: a systematic review and meta-analysis of cohort studies

**DOI:** 10.1136/bjsports-2021-105061

**Published:** 2022-02-28

**Authors:** Haruki Momma, Ryoko Kawakami, Takanori Honda, Susumu S Sawada

**Affiliations:** 1 Department of Medicine and Science in Sports and Exercise, Tohoku University Graduate School of Medicine, Sendai, Miyagi, Japan; 2 Faculty of Sport Sciences, Waseda University, Tokorozawa, Saitama, Japan; 3 Department of Epidemiology and Public Health, Kyushu University Graduate School of Medical Sciences, Fukuoka, Japan

**Keywords:** meta-analysis, cardiovascular diseases, weight lifting, survival, cohort studies

## Abstract

**Objective:**

To quantify the associations between muscle-strengthening activities and the risk of non-communicable diseases and mortality in adults independent of aerobic activities.

**Design:**

Systematic review and meta-analysis of prospective cohort studies.

**Data sources:**

MEDLINE and Embase were searched from inception to June 2021 and the reference lists of all related articles were reviewed.

**Eligibility criteria for selecting studies:**

Prospective cohort studies that examined the association between muscle-strengthening activities and health outcomes in adults aged ≥18 years without severe health conditions.

**Results:**

Sixteen studies met the eligibility criteria. Muscle-strengthening activities were associated with a 10–17% lower risk of all-cause mortality, cardiovascular disease (CVD), total cancer, diabetes and lung cancer. No association was found between muscle-strengthening activities and the risk of some site-specific cancers (colon, kidney, bladder and pancreatic cancers). J-shaped associations with the maximum risk reduction (approximately 10–20%) at approximately 30–60 min/week of muscle-strengthening activities were found for all-cause mortality, CVD and total cancer, whereas an L-shaped association showing a large risk reduction at up to 60 min/week of muscle-strengthening activities was observed for diabetes. Combined muscle-strengthening and aerobic activities (versus none) were associated with a lower risk of all-cause, CVD and total cancer mortality.

**Conclusion:**

Muscle-strengthening activities were inversely associated with the risk of all-cause mortality and major non-communicable diseases including CVD, total cancer, diabetes and lung cancer; however, the influence of a higher volume of muscle-strengthening activities on all-cause mortality, CVD and total cancer is unclear when considering the observed J-shaped associations.

**Systematic review registration:**

PROSPERO CRD42020219808.

## Introduction

Physical inactivity is a global public health problem. Several national and international physical activity guidelines recommend regular muscle-strengthening activities for adults.^1–5^ For example, the recent WHO guidelines recommend that adults should perform muscle-strengthening activities ≥2 days/week.^4^ Regular engagement in muscle-strengthening activities (eg, resistance training) increases or preserves skeletal muscle strength,^3^ which has been shown to be inversely associated with mortality^6 7^ and the risk of non-communicable diseases (NCDs) such as cardiovascular disease (CVD) and cancer.^7^ Therefore, promoting muscle-strengthening activities may help in reducing the risk of premature death and NCDs.

Compared with aerobic activities, muscle-strengthening activities have been less frequently investigated in terms of their influence on the prevention of premature death and NCDs. Saeidifard *et al* conducted the first systematic review and meta-analysis of 11 published studies that focused on mortality.^8^ Although no clear association was observed between resistance training and mortality from CVD and cancer, resistance training was found to be inversely associated with all-cause mortality.^8^ Moreover, a recent meta-analysis that focused on cancer incidence and mortality showed that muscle-strengthening activities were associated with a lower incidence of kidney cancer.^9^ Although these findings suggested a favourable influence of muscle-strengthening activities on the risk of NCDs and mortality, the dose–response association was not quantified. In some countries such as Japan,^10^ a revision of the national physical activity guidelines is under way, and there is a debate regarding whether muscle-strengthening activities should be included in the guidelines. Existing physical activity guidelines primarily focus on the musculoskeletal health benefits of muscle-strengthening activities.^11–13^ A systematic evaluation of the associations of muscle-strengthening activities with mortality and NCDs will aid in determining whether muscle-strengthening activities need to be included in the guidelines. In addition, investigating the dose–response association is also necessary to determine the amount of muscle-strengthening activities that should be recommended for public health purposes. A recent narrative review suggested the existence of dose–response associations between muscle-strengthening activities and mortality and major NCDs.^14^ With the increasing number of relevant cohort studies, it is now possible to systematically update and expand on previous reviews that did not directly provide the optimal dose of muscle-strengthening activities.

We therefore conducted a systematic review and meta-analysis of prospective cohort studies on muscle-strengthening activities and the risk of mortality and NCDs among adults aged ≥18 years. In addition to examining the health benefits of engaging in muscle-strengthening activities compared with the absence of muscle-strengthening activities independent of aerobic activities, we quantified the dose–response association between muscle-strengthening activities and health outcomes. We also focused on the additional benefits of combined muscle-strengthening and aerobic activities for health outcomes.

## Methods

This systematic review was performed following the MOOSE^15^ and PRISMA 2020^16^ guidelines and was registered a priori in the PROSPERO database (CRD42020219808).

### Data sources and searches

A systematic literature search was conducted in MEDLINE and Embase from the inception of the databases to 25 October 2020. The search syntax was designed by professional research agencies (International Medical Information Centre, Tokyo, Japan and Inforesta Co Ltd, Tokyo, Japan) with input from two authors (HM and RK) (see online supplemental table 1). We focused on the literature on the association between muscle-strengthening activities and health outcomes among adults aged ≥18 years without diagnosed severe health conditions (eg, cancer or disability) at baseline. Studies were considered eligible if they (1) had a prospective observational design; (2) had a minimum follow-up period of 2 years; (3) examined the influence of muscle-strengthening activities on the outcomes independent of and in combination with aerobic activities; and (4) were published in English. We included studies that used any health outcomes except for those that used a surrogate marker as an outcome.

10.1136/bjsports-2021-105061.supp1 Supplementary data

### Study selection

To select articles for full-text reading, two authors (HM and RK) independently screened the titles and abstracts using EndNote X9.2 (Clarivate Analytics, Pennsylvania, USA) and Microsoft Excel (Microsoft Corporation, Redmond, Washington, USA) after the exclusion of duplicates. Articles with ambiguous eligibility were included in the full-text reading step. The two authors also independently performed full-text reading of each article and a hand-search of the reference lists in the selected articles. No additional studies were found. Disagreements were resolved through discussion. An update of the primary search was conducted in June 2021.

### Data extraction

Three authors (HM, RK, and TH) independently extracted the following information from each eligible study after dividing the selected papers among them: first author, publication year, study location, cohort name, sex, age of participants, number of participants and person-years, years of follow-up, number of deaths, cause of death, number of incident outcomes, subtype of incident outcome, assessment details for outcomes, assessment details for muscle-strengthening activities, covariates included in the analyses, and effect estimates and 95% confidence intervals (CIs) of mortality or incidence of NCDs. If relevant information about the assessment of outcomes and exposures was missing from the eligible studies, we obtained the information from other studies of the same cohort. The most adjusted effect estimates in the main and sensitivity analyses were extracted. For each study, one of the three authors extracted the data and the remaining two authors cross-checked the data. Disagreements were resolved through deliberation to achieve consensus. Because most of the studies eligible for our meta-analyses reported hazard ratios, if other effect estimates such as ORs were reported, we asked the corresponding authors to provide the hazard ratios.^17 18^ Moreover, if information about the effect estimate was not reported, we asked the corresponding authors to provide the hazard ratios using a template.^19–21^ Three authors provided additional data.^17 19 20^ When multiple articles involving the same cohort for the same outcome were identified, only data from the most recently published article were used. In all such cases, the most recently published articles had the largest number of cases in our systematic review. When the publication year was the same, the article with the largest number of participants and cases was included.

### Quality assessment

The quality of the studies was assessed using a modification of the Newcastle–Ottawa Scale (NOS) for Quality Assessment of Prospective Cohort Studies (see online supplemental table 2).^22^ We excluded the ‘representativeness of the exposed cohort’ item of the original NOS because our quality assessment was planned to evaluate internal validity, not external validity. Therefore, 8 stars in total were achievable, and a higher score indicated higher study quality. HM and RK independently assessed the studies and resolved any inconsistencies through discussion.

### Data synthesis and analysis

A meta-analysis was conducted if at least two studies reported the effect estimate for the same outcome. Reported hazard ratios were considered equivalent to relative risks (RRs). When only ORs were available,^18^ they were considered equivalent to RRs because the overall cumulative incidence of the outcome was relatively low (16.5%). Although we tried to convert ORs to RRs, we could not obtain an assumed control risk from the study because the number of cases was not provided. We assessed the influence of the inclusion of this study by performing a leave-one-out analysis. For the meta-analysis of the influence of muscle-strengthening activities, the effect estimates for any muscle-strengthening activities compared with no muscle-strengthening activities were combined using the random-effects model of DerSimonian and Laird.^23^ When the included studies had two or more exposed groups, the effect estimates among the exposed groups were synthesised to obtain a pooled effect estimate using a fixed-effects model with the inverse variance method.^24 25^

We also conducted a dose–response meta-analysis to investigate the influence of muscle-strengthening activities on health outcomes using the method described by Greenland and Longnecker^26^ and Orsini *et al*.^27^ This method allows estimating study-specific linear trends (slopes) considering the covariance for each exposure category within each study because they are calculated relative to a common reference group.^26 27^ The method requires data including distribution of cases, person-years and adjusted RR with 95% CI across three or more quantitative categories. If only the total number of cases or person-years was reported, the distribution of cases or person-years was estimated using the total number of cases and person-years and the RR according to the previous study.^28^ If the total number of person-years was not reported, we approximated it by multiplying the total number of participants by the median or mean of the follow-up period. The median or mean of the time of muscle-strengthening activities within the exposure categories was assigned to the corresponding RR. If these were not reported, the midpoint between the lower and upper limits was calculated. For open-ended categories, we assumed that they had the same widths as the closest category. We used ‘none’ as the reference group, and there was no study in which the reference category was not the lowest category. The study-specific slopes were pooled using the DerSimonian and Laird random-effects model.^23^ A potential non-linear association was also examined using a restricted cubic spline model with three knots at fixed percentiles (10%, 50% and 90%) of time of the exposure.^29^ Non-linearity was assessed by testing the null hypothesis that the coefficient of the second spline was equal to zero using a Wald test.^29^

The joint benefit of muscle-strengthening activities and aerobic activities was also examined using the studies that reported the effect estimates of both muscle-strengthening and aerobic activities. The categories of muscle-strengthening (eg, none vs any or ≥2 vs <2 times/week) and aerobic activity (eg, ≥150 vs <150 min/week or low vs high) were defined on the basis of the included studies.

Statistical heterogeneity between studies was examined using Cochrane’s Q test and I^2^ statistic. I^2^ statistic with values of 25%, 50% and 75% corresponded to low, moderate and high level of heterogeneity, respectively.^30^ To examine the effect of individual studies on the pooled point estimate and 95% CI of each outcome, we performed a sensitivity analysis by serially excluding each study and evaluated the corresponding changes in the effect estimate (leave-one-out analysis).

Subgroup analyses were performed according to sex (men only, women only, or men and women), age (>65 or ≤65 years), exposure assessment (post hoc, questionnaire or interview) and NOS quality score (post hoc, <7 or ≥7). However, subgroup analyses according to age and sex with cancer as the outcome were not performed owing to insufficient data.

Publication bias was assessed by visually inspecting the funnel plots of estimates against the SE of each study and by using Egger’s test of funnel plot asymmetry^31^ if the number of included studies was ≥10.^32^

All analyses were performed using Stata 17 (StataCorp, College Station, Texas, USA). Statistical significance was set at p<0.05.

### Grading the evidence

The Grading of Recommendations Assessment, Development and Evaluation (GRADE) approach was used to assess the overall certainty of evidence for outcomes.^33–38^ One reviewer (HM) assessed the certainty of the evidence while two reviewers (RK and TH) examined and revised the certainty of assessments as necessary. A GRADE evidence profile was developed (see online supplemental table 3).^39^

## Results

### Literature search

**Figure 1:**
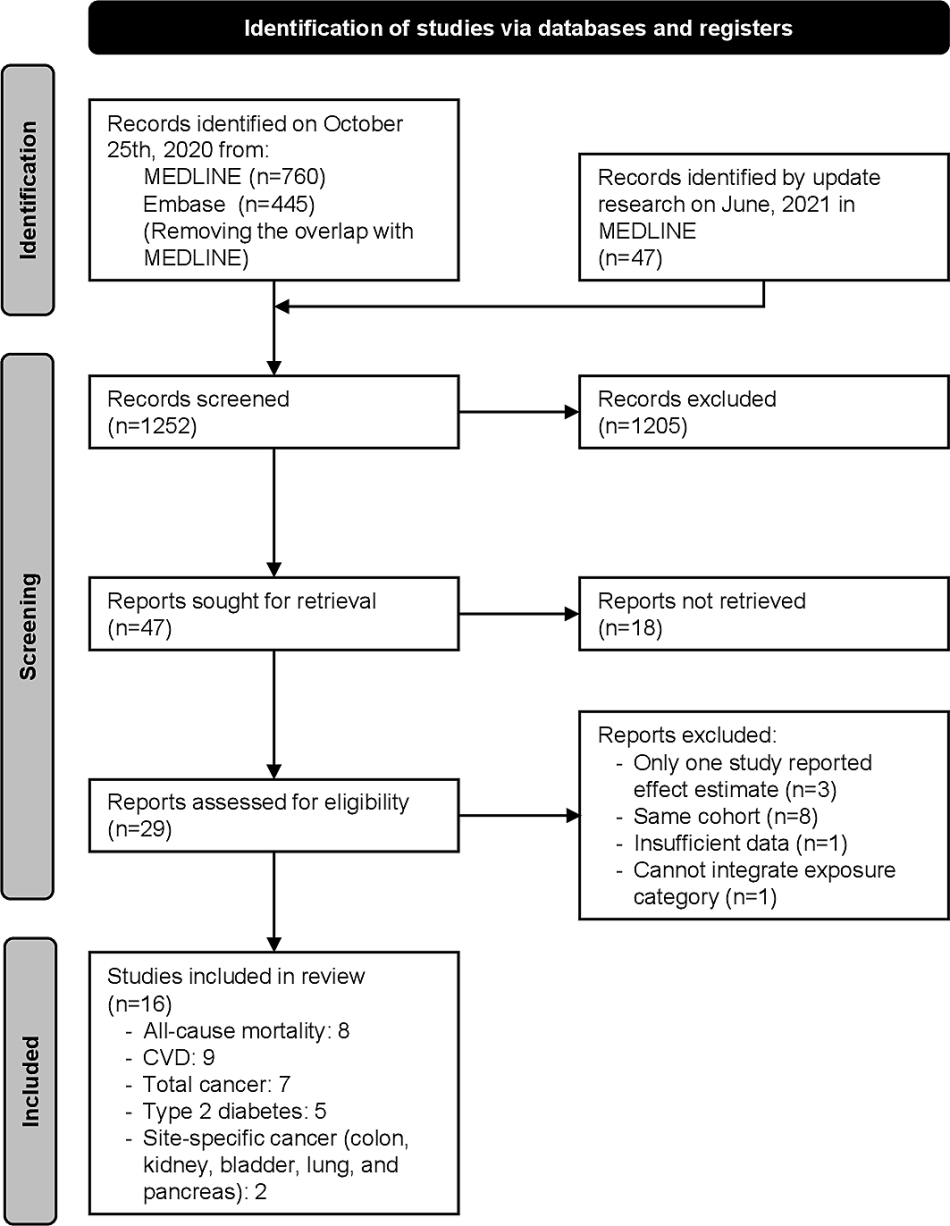
Flowchart of the selection of studies included in the meta-analysis.

A total of 1252 records were identified through systematic searches in MEDLINE and Embase after the removal of duplicates. Of these, 47 records were retrieved for full-text review and 29 studies were eligible based on the inclusion criteria.^17–21 40–63^ Among them, although a total of 28 outcomes were reported, only nine outcomes (all-cause mortality, CVD, total cancer, diabetes and site-specific cancers (colon, kidney, bladder, lung and pancreatic cancers)) were examined in two or more studies. Therefore, 17 outcomes were excluded from our meta-analyses (see online supplemental table 4), resulting in the exclusion of three studies.^60–62^ Moreover, prostate cancer and lymphoma were also excluded because of discrepancies in the definition of outcomes across the studies.^45 50^ Of the remaining 26 studies we excluded eight because of multiple publications from the same cohort (see online supplemental table 5).^52–59^ One study was further excluded because of insufficient information about the effect estimate^21^ and another study was excluded because the exposure could not be integrated.^63^ Finally, 16 studies were included in the meta-analysis (figure 1).^17–20 40–51^

### Study characteristics

The detailed characteristics of the studies included in the meta-analysis are presented in online supplemental table 6. The publication years ranged from 2012 to 2020. Most studies were conducted in the USA.^18 19 40 42–51^ Other studies were from England and Scotland,^20^ Australia^17^ and Japan.^41^ The number of participants varied considerably (from 3809 to 479 856). The maximum follow-up duration was 25.2 years (median).^46^ The age of participants ranged from 18 to 97.8 years. Twelve studies included both men and women,^17 18 20 41 44–49 51^ two studies included men only^19 50^ and three studies included women only.^40 42 43^ Adjustment for confounders varied widely across studies, with most studies adjusting for age, body mass index, alcohol intake and smoking status, whereas several studies adjusted for sex, race/ethnicity, dietary habits, disease history and sociodemographic status. All studies considered aerobic or other types of physical activity. Thirteen studies used self-reporting methods to measure muscle-strengthening activities^17–20 40–46 48 50^ and three studies used interview methods.^47 49 51^ All studies focused on muscle-strengthening exercises such as resistance/strength/weight training and callisthenics, but not on muscle-strengthening activities such as carrying heavy loads and heavy gardening.

### Risk of bias and certainty of evidence

In the risk of bias assessment using the NOS (online supplemental table 2), the included studies were assigned 4–7 stars. For all-cause mortality, four studies were assigned 7 stars, three studies were assigned 6 stars and one study was assigned 5 stars. For CVD, four studies were assigned 7 or 6 stars whereas one study was assigned 5 stars. For total cancer, four and three studies were assigned 7 and 6 stars, respectively, whereas one study was assigned 5 stars. For diabetes, four studies were assigned 6 stars and one study was assigned 4 stars.

The overall certainty of the evidence for each outcome and its details are shown in table 1 and online supplemental table 3. The grading of the certainty of the evidence was generally very low. The main reason for downgrading the evidence was indirectness because most of the studies included in this review were conducted in the USA.

**Table 1:** Summary of the association between muscle-strengthening activities and health outcomes

Outcomes	Two-group (no vs any muscle-strengthening activities) meta-analysis					Dose–response meta-analysis (10 min/week increase)					
	N	Cases/participants	RR (95% CI)	P value	I^2^, p value	N	Cases/participants	RR (95% CI)	P value	I^2^, p value	GRADE*
All-cause mortality	7	42 133/263 058	0.85 (0.79 to 0.93)	<0.001	83%,<0.001	6	37 178/236 331	0.99 (0.98 to 1.00)†	0.05	75%, 0.001	⨁◯◯◯
CVD	7	16 056/257 888	0.83 (0.73 to 0.93)	0.002	73%,<0.001	5	11 263/226 746	0.996 (0.99 to 1.003)‡	0.26	0%, 0.46	⨁◯◯◯
Total cancer	6	21 253/540 543	0.88 (0.80 to 0.97)	0.008	76%,<0.001	4	13 033/212 323	0.99 (0.98 to 1.004)§	0.15	80%, 0.002	⨁◯◯◯
Diabetes	5	9548/202 486	0.83 (0.77 to 0.89)	<0.001	36%, 0.18	3	7511/167 072	0.98 (0.97 to 0.99)¶	0.003	59%, 0.09	⨁⨁◯◯
Colon cancer	2	2415/248 909	0.96 (0.91 to 1.01)	0.09	0%,<0.35	2	2415/248 909	0.998 (0.96 to 1.04)	0.91	94%,<0.001	⨁◯◯◯
Kidney cancer	2	1063/248 909	0.88 (0.76 to 1.02)	0.08	0%,<0.52	2	1063/248 909	0.98 (0.96 to 1.002)	0.08	9%, 0.29	⨁◯◯◯
Bladder cancer	2	2341/248 909	0.94 (0.84 to 1.05)	0.27	19%,<0.27	2	2341/248 909	0.98 (0.95 to 1.02)	0.34	77%, 0.04	⨁◯◯◯
Lung cancer	2	4075/248 909	0.90 (0.83 to 0.98)	0.01	0%,<0.69	2	4075/248 909	0.99 (0.98 to 1.00)	0.045	0%, 0.81	⨁◯◯◯
Pancreatic cancer	2	1028/248 909	1.12 (0.98 to 1.28)	0.11	0%,<0.84	2	1028/248 909	1.004 (0.99 to 1.02)	0.65	0%, 0.89	⨁◯◯◯

*⨁◯◯◯: very low; ⨁⨁◯◯: low; ⨁⨁⨁◯: moderate; ⨁⨁⨁⨁: high.

†A J-shaped association with the maximum risk reduction (17%) at 40 min/week.

‡A J-shaped association with the maximum risk reduction (18%) at 60 min/week.

§A J-shaped association with the maximum risk reduction (9%) at 30 min/week.

¶An L-shaped association with a large risk reduction up to 60 min/week.

CVD, cardiovascular diseases; GRADE, Grading of Recommendations Assessment, Development and Evaluation; RR, relative risk.

### All-cause mortality

**Figure 2:**
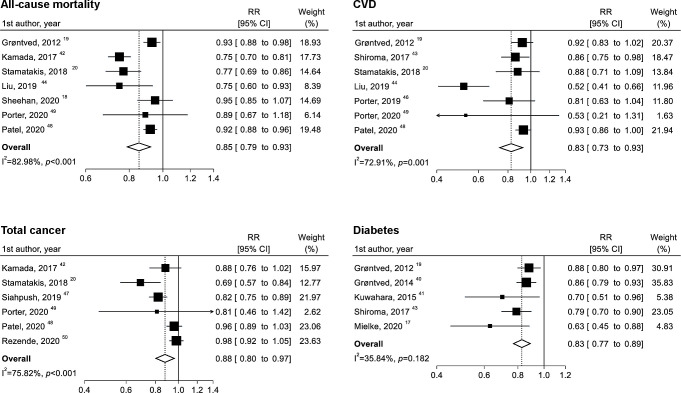
Two-group meta-analysis of the associations between no versus any muscle-strengthening activities and all-cause mortality, cardiovascular disease (CVD), total cancer and diabetes. RR, relative risk.

Seven studies with 42 133 cases of all-cause mortality among 263 058 participants were included in the two-group analysis. Muscle-strengthening activities were associated with a 15% lower risk of all-cause mortality (RR 0.85; 95% CI 0.79 to 0.93; p<0.001) (figure 2). Although the heterogeneity was high (I^2^=83.0%; p<0.001), the association was in the same direction, with an RR of <1.00 in all studies. A similar result was obtained when Sheehan’s study,^18^ which provided ORs, was excluded (RR 0.84; 95% CI 0.76 to 0.92; p<0.001) (see online supplemental figure 1). Moreover, the exclusion of any other individual study did not substantially change this result, and the high heterogeneity was not explained by sex, quality score or exposure assessment (see online supplemental figures 1-4).

**Figure 3:**
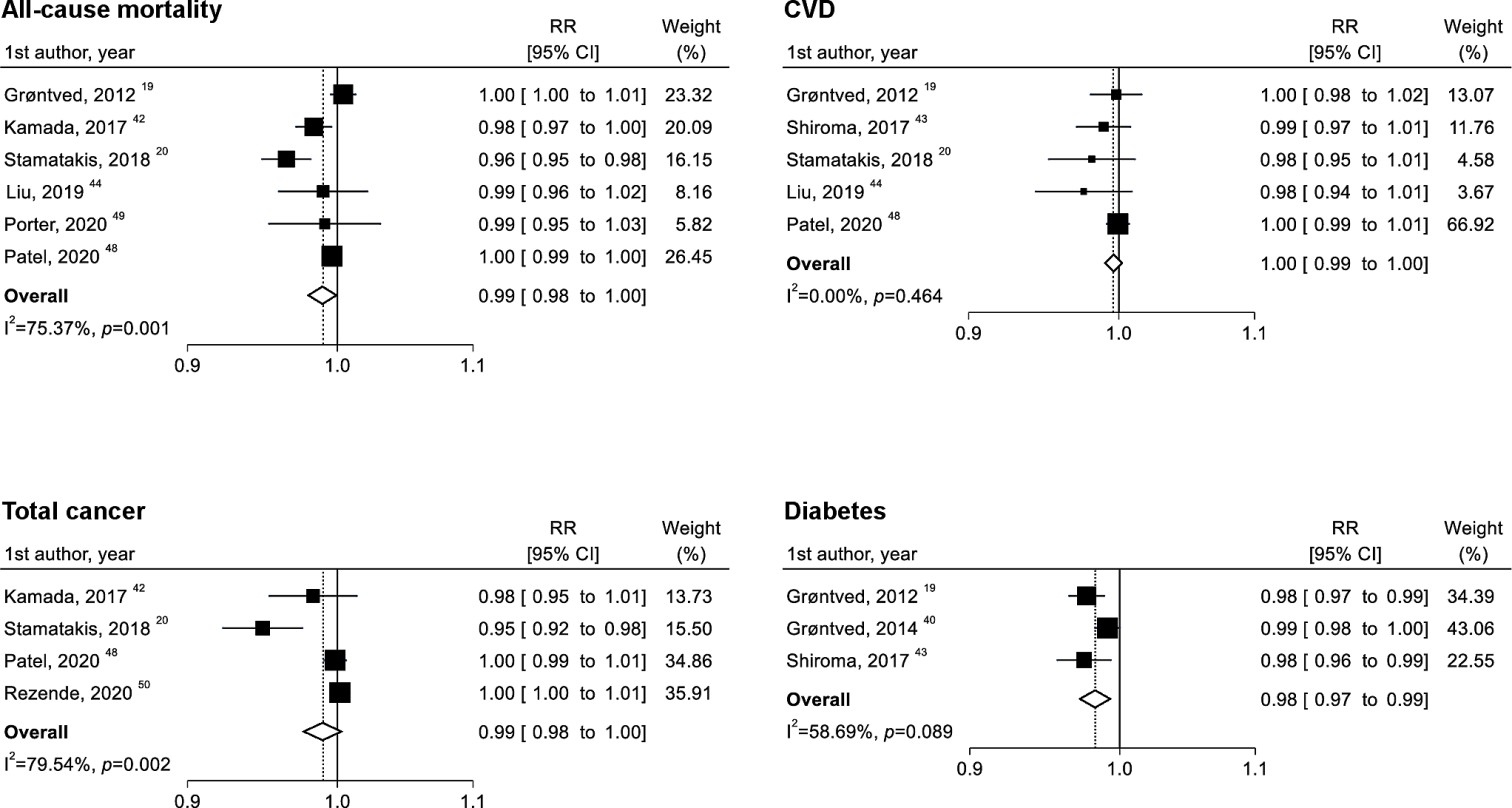
Linear dose–response meta-analysis of the associations between muscle-strengthening activities (per 10 min/week increase) and all-cause mortality, cardiovascular disease (CVD), total cancer and diabetes. RR, relative risk.

**Figure 4:**
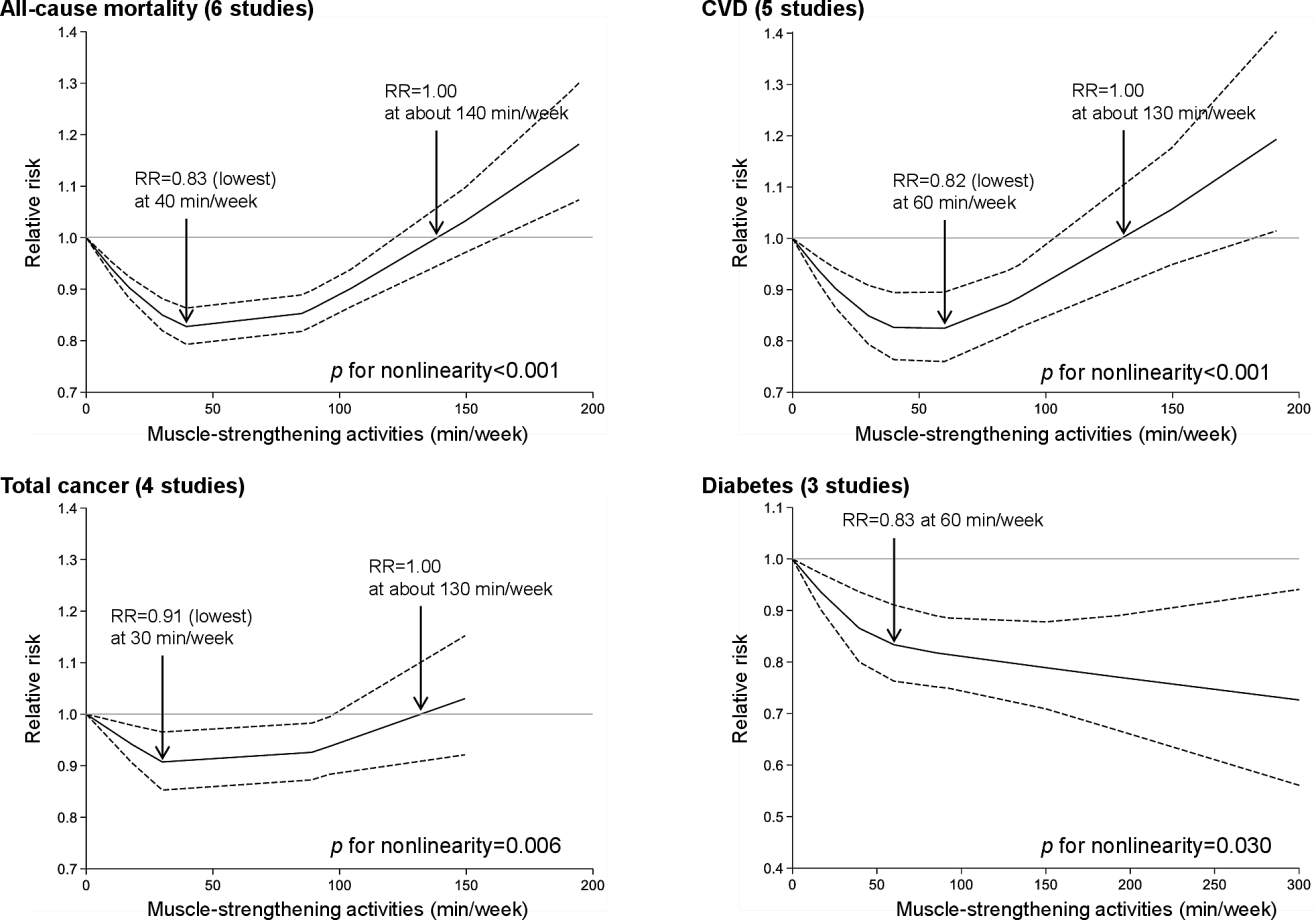
Non-linear dose–response meta-analysis of the associations between muscle-strengthening activities and all-cause mortality, cardiovascular disease (CVD), total cancer and diabetes. Muscle-strengthening activities were modelled with restricted cubic splines in a random-effects dose–response model. The black line indicates the spline model and dashed lines represent 95% confidence intervals. RR, relative risk.

Six studies were eligible for the dose–response analysis of muscle-strengthening activities per 10 min/week increase, with a total of 236 331 participants and 37 178 cases. Although there was no clear linear association (figure 3), a non-linear association was observed (figure 4). The lowest RR (RR 0.83; 95% CI 0.79 to 0.86) was observed at 40 min/week of muscle-strengthening activities, and the RR estimate for up to approximately 140 min/week was <1.00.

**Figure 5:**
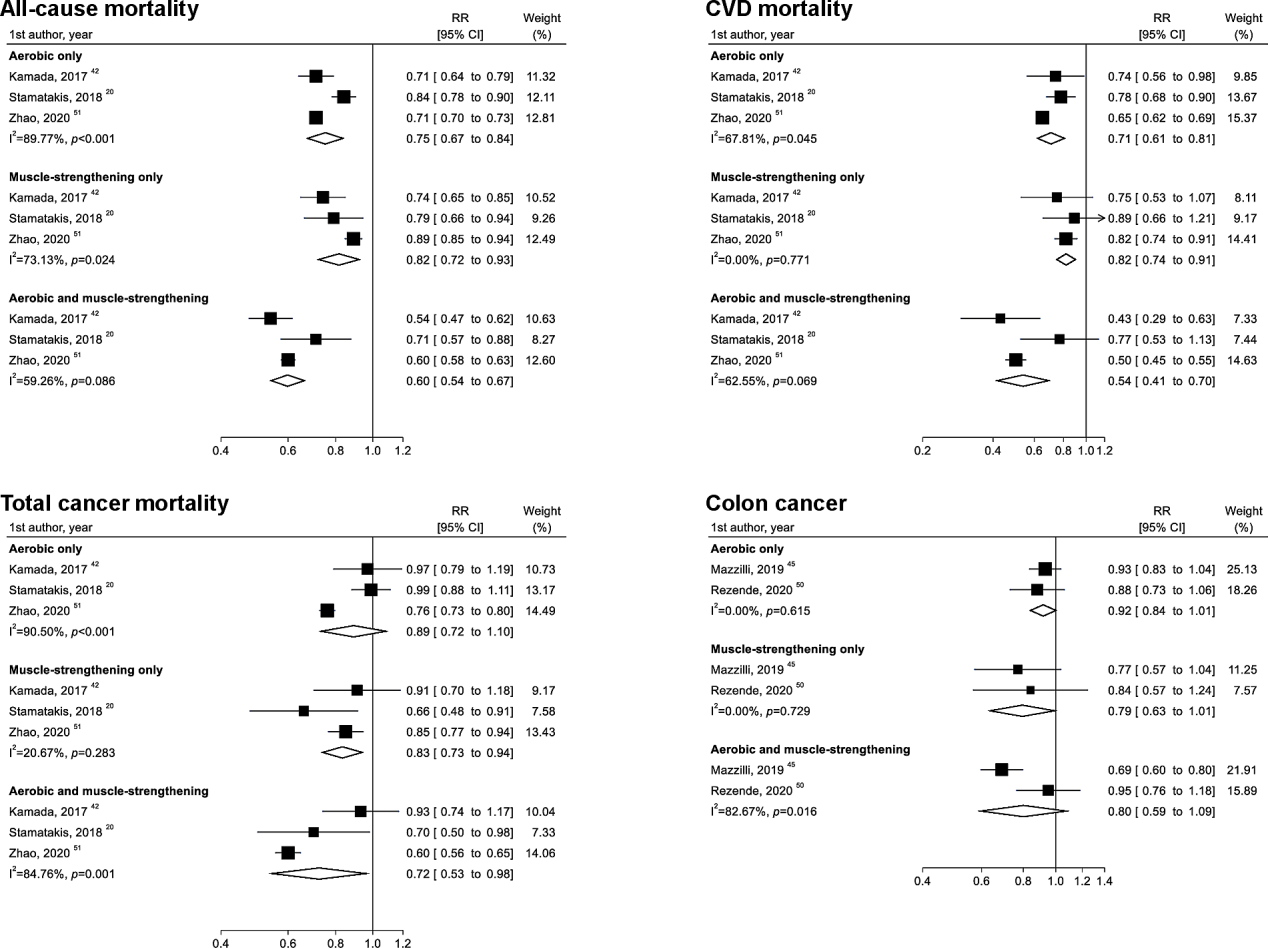
Meta-analysis of the joint associations of muscle-strengthening and aerobic activities with all-cause mortality, cardiovascular disease (CVD) mortality, total cancer mortality and colon cancer incidence. The definitions of groups for muscle-strengthening and aerobic activities were based on the categories described in online supplemental table 6. RR, relative risk.

Three studies examined the joint benefit of muscle-strengthening and aerobic activities for all-cause mortality, with a total of 581 194 participants and 68 637 cases. Combined muscle-strengthening and aerobic activities (vs none) were associated with a 40% lower risk of all-cause mortality (RR 0.60; 95% CI 0.54 to 0.67; I^2^=59.3%) (figure 5).

The overall quality of the evidence on all-cause mortality was rated as ‘very low’.

### CVD

Seven studies with 16 056 cases of CVD among 257 888 participants were included in the two-group analysis. Three studies focused on CVD mortality or CVD morbidity,^43 44 46^ whereas other studies focused on CVD mortality.^19 20 42 48 49 51^ Muscle-strengthening activities were associated with a 17% lower risk of CVD (RR 0.83; 95% CI 0.73 to 0.93; p=0.002), with a high level of heterogeneity (I^2^=72.9%; p=0.001) (figure 2). Although the high heterogeneity was not completely explained by the quality score and exposure assessment, the heterogeneity disappeared (I^2^=0.0%) when the study by Liu *et al* 44 was excluded (online supplemental figure 1). Moreover, a similar result was obtained when the analysis was limited to CVD mortality (online supplemental figure 5).

Five studies were eligible for the dose–response analysis of muscle-strengthening activities per 10 min/week increase, with a total of 226 746 participants and 11 263 cases. Although there was no clear linear association (figure 3), a non-linear association was observed (figure 4). The lowest RR (RR 0.82; 95% CI 0.76 to 0.90) was observed at 60 min/week of muscle-strengthening activities, and the RR estimate for up to approximately 130 min/week was <1.00.

Three studies examined the joint benefit of muscle-strengthening and aerobic activities for CVD mortality, with a total of 582 672 participants and 15 643 cases. Combined muscle-strengthening and aerobic activities were associated with a 46% lower risk of CVD (RR 0.54; 95% CI 0.41 to 0.70; I^2^=62.6%) (figure 5).

The overall quality of the evidence on CVD was rated as ‘very low’.

### Total cancer

Six studies with 21 253 cases of total cancer among 540 543 participants were included in the two-group analysis. One study focused on total cancer incidence,^50^ whereas the other studies focused on total cancer mortality.^20 42 47–49^ Muscle-strengthening activities were associated with a 12% lower risk of total cancer (RR 0.88; 95% CI 0.80 to 0.97; p=0.008), with a high level of heterogeneity (I^2^=75.8%; p<0.001) (figure 2). The exclusion of any individual study did not substantially change this result, and the high heterogeneity was not explained by the quality score or exposure assessment (online supplemental figures 1-3). When the analysis was limited to total cancer mortality (ie, excluding the study by Rezende *et al* 50), a similar result was obtained (online supplemental figure 1).

Four studies were eligible for the dose–response analysis of muscle-strengthening exercise per 10 min/week increase, with a total of 212 323 participants and 13 033 cases. Although there was no linear association (figure 3), a non-linear association was observed (figure 4). The lowest RR (RR 0.91; 95% CI 0.85 to 0.97) was observed at 30 min/week of muscle-strengthening activities and the RR estimate for up to approximately 130 min/week was <1.00.

Three studies examined the joint benefit of muscle-strengthening and aerobic activities for total cancer mortality, with a total of 585 930 participants and 17 212 cases. Combined muscle-strengthening and aerobic activities were associated with a 28% lower risk of total cancer mortality (RR 0.72; 95% CI 0.53 to 0.98; I^2^=84.8%) (figure 5).

The overall quality of the evidence on total cancer was rated as ‘very low’.

### Diabetes

Five studies with 9548 cases of diabetes among 202 486 participants were included in the two-group analysis. Muscle-strengthening activities were associated with a 17% lower incidence of diabetes (RR 0.83; 0.77 to 0.89; p<0.001), with a low to moderate level of heterogeneity (I^2^=35.8%; p=0.18) (figure 2). The heterogeneity was substantially reduced (I^2^=9.5%) when the study by Mielke *et al* 17 with low quality (NOS=4) was excluded (online supplemental figure 1). An inverse association was obtained when the analysis was limited to studies focused on women (two studies) (online supplemental figure 5).

Three studies were eligible for the dose–response analysis of muscle-strengthening activities per 10 min/week increase, with a total of 167 072 participants and 7511 cases. Each 10 min/week increase in muscle-strengthening activities was inversely associated with the risk of diabetes, with moderate evidence of heterogeneity (RR 0.98; 95% CI 0.97 to 0.99; p=0.003; I^2^=58.7%; p=0.09) (figure 3). Moreover, an L-shaped relationship was found, and the risk markedly decreased until up to 60 min/week of muscle-strengthening activities (figure 4).

The overall quality of the evidence on diabetes was rated as ‘low’.

### Site-specific cancers

Two studies were included in the two-group and dose–response analyses for the incidence of site-specific cancers (colon, kidney, bladder, lung and pancreatic cancers).^45 50^ The total number of cases/participants was 2415/248 909 for colon cancer, 1063/248 909 for kidney cancer, 2341/248 909 for bladder cancer, 4075/248 909 for lung cancer and 1028/248 909 for pancreatic cancer. Muscle-strengthening activities were associated with a 10% lower incidence of lung cancer (RR 0.90; 95% CI 0.83 to 0.98; p=0.01; I^2^=0.0%; p=0.69) (online supplemental figure 6). A linear association was obtained for lung cancer (RR 0.99; 95% CI 0.98 to 1.00; p=0.045; I^2^=0.0%; p=0.81) (online supplemental figure 7). For other site-specific cancers, no association was confirmed in the two-group, dose–response and joint analyses (figure 5 and online supplemental figures 6 and 7).

Sensitivity analysis and any subgroup analysis were not performed because of the small number of included studies.

The overall quality of the evidence on the incidence of each site-specific cancer was rated as ‘very low’.

### Publication bias

For all outcomes included in the meta-analysis, the test for funnel plot asymmetry was not performed because of the small number of included studies (n≤7).

## Discussion

This systematic review and meta-analysis of cohort studies found that muscle-strengthening activities were inversely associated with the risk of CVD, total cancer, diabetes, lung cancer and all-cause mortality independent of aerobic activities among adults aged ≥18 years without severe health conditions. Moreover, J-shaped associations were found between muscle-strengthening activities and all-cause mortality, CVD and total cancer, with the maximum risk reduction (approximately 10–20%) at approximately 30–60 min/week of muscle-strengthening activities. We also observed an L-shaped association between muscle-strengthening activities and diabetes, showing a large risk reduction before 60 min/week. Finally, combined muscle-strengthening and aerobic activities (vs none) were associated with a lower risk of all-cause, CVD and total cancer mortality.

Saeidifard *et al* reported that engaging in muscle-strengthening activities was associated with a lower risk of all-cause mortality, although there was no clear association with CVD mortality and total cancer mortality.^8^ Moreover, another meta-analysis showed no clear association with total cancer mortality.^9^ Our systematic review updated the literature and expanded on previous studies,^8 9^ showing that muscle-strengthening activities were inversely associated with the risk of CVD, total cancer and all-cause mortality. We obtained similar results when the analysis was limited to CVD and total cancer mortality. In addition, muscle-strengthening activities were associated with a lower incidence of lung cancer in our review, although Nascimento *et al* showed an inverse association for kidney cancer, but not lung cancer, even when the same studies were included.^9^ The reason for this discrepancy may be derived from the extracted effect estimates. Nascimento *et al* extracted the effect estimate from the highest category of muscle-strengthening activities whereas we used pooled effect estimates when the included studies had two or more exposed groups.

Joint analysis between muscle-strengthening and aerobic activities showed that a greater benefit for all-cause, CVD and total cancer mortality was obtained when these two types of activities were combined. These results confirm the findings of previous meta-analyses.^8 9^ Therefore, beyond aerobic activities, muscle-strengthening activities may provide additional benefits for preventing mortality.

One of the strengths of this study was the quantification of the dose–response association between muscle-strengthening activities and health outcomes. Several previous cohort studies have reported a non-linear association between muscle-strengthening activities and health outcomes.^42–44 48^ For example, Kamada *et al* showed a quadratic association between strength training and all-cause and CVD mortality, and the lowest risk of all-cause mortality was observed at 82 min/week of strength training.^42^ Furthermore, the abovementioned previous meta-analysis reported that performing resistance training 1–2 times/week was associated with a lower all-cause mortality, but increasing the volume to >2 times/week was not.^8^ This result supports a potential non-linear association between muscle-strengthening activities and all-cause mortality. In our systematic review, J-shaped associations with the maximum risk reduction (10–20%) at approximately 30–60 min/week of muscle-strengthening activities were observed for all-cause mortality, CVD and total cancer. These results suggest that optimal doses of muscle-strengthening activities for the prevention of all-cause death, CVD and total cancer may exist.

In addition, our study is the first to systematically evaluate the longitudinal association between muscle-strengthening activities and the risk of diabetes. Although the potential of muscle-strengthening activities to reduce the risk of diabetes is supported by several biological mechanisms,^64 65^ many of the previous studies on this topic were limited to short-term randomised controlled trials examining surrogates of diabetes.^66^ Our findings showed that muscle-strengthening activities were associated with a 17% lower incidence of diabetes, with the risk of diabetes sharply decreasing until up to 60 min/week of muscle-strengthening activities followed by a gradual decrease. Because muscle-strengthening activities increase or preserve skeletal muscle mass, which has been identified as the major tissue in glucose metabolism, a clear dose–response association can be established.

Our systematic review has some limitations. The first and most important limitation is that the meta-analysis included only a small number of studies. The limited number of studies precluded some examinations. For example, it did not allow us to conduct some subgroup analyses to explain the heterogeneity in our findings and, even when performed, few studies were included. Moreover, we could not test for publication bias. Therefore, the pooled estimates in this study might have been overestimated because of potential publication bias. Second, the included studies evaluated muscle-strengthening activities using a self-reported questionnaire or the interview method. Although measures of muscle-strengthening activities have been reported to have higher reliability than those of aerobic activities,^67^ this may have contributed to the heterogeneity in our results. Indeed, the heterogeneities in this review were partially explained by differences in exposure assessment, although only a few studies were included. Third, because most of the included studies were conducted in the USA, the generalisability of our findings is limited. Fourth, observational studies were included in the meta-analysis and were thus potentially influenced by residual, unknown and unmeasured confounding factors. Finally, only two databases were searched, and therefore some relevant studies may have been missed.

Several physical activity guidelines recommend that adults perform muscle-strengthening activities at least twice a week.^1–5^ Although the recommendation is primarily based on the benefit for musculoskeletal health,^11–13^ these guidelines are partly supported by our results in terms of preventing premature death and NCDs. However, the influence of a higher volume of muscle-strengthening activities on health benefits is unclear. Our findings showed that the maximum risk reduction for all-cause mortality, CVD and total cancer was obtained at approximately 30–60 min/week of muscle-strengthening activities, and the RR was low for up to approximately 130–140 min/week. Given this result, the current recommendation of at least 2 days/week could be reasonable, although a higher volume may require caution. However, our findings should be interpreted with caution because the number of included studies was small and we could not directly examine the frequency of muscle-strengthening activities. Large-scale studies are needed to examine the health benefits of high-volume muscle-strengthening activities. Moreover, attention should also be paid to evidence that most programmes providing benefits for musculoskeletal health in elderly people are performed ≥2 days/week.^12^ The longitudinal influence of muscle-strengthening activities on mortality and NCDs should be further investigated with a focus on the elderly population in future studies.

## Conclusion

Engaging in muscle-strengthening activities was associated with a lower risk of all-cause mortality and major NCDs such as CVD, total cancer, diabetes and lung cancer. However, the influence of a higher volume of muscle-strengthening activities on all-cause mortality, CVD and total cancer is unclear, considering the observed J-shaped associations. In addition, the combination of muscle-strengthening and aerobic activities may provide a greater benefit for reducing all-cause, CVD and total cancer mortality. Given that the available data are limited, further studies—such as studies focusing on a more diverse population—are needed to increase the certainty of the evidence.

**What is already known?**

- Physical activity guidelines recommend regular muscle-strengthening activities for adults, and this recommendation is primarily based on the benefits for musculoskeletal health.
- Previous meta-analyses have shown that muscle-strengthening activities are associated with a decreased risk of all-cause mortality and kidney cancer, although the dose–response association is unknown.
- Further studies are needed to update the literature and expand on previous studies that did not provide evidence on the optimal dose of muscle-strengthening activities.

**What are the new findings?**

- Muscle-strengthening activities were associated with a 10–17% lower risk of CVD, total cancer, diabetes, lung cancer and all-cause mortality independent of aerobic activities among adults.
- The maximum risk reduction for all-cause mortality, CVD and total cancer was obtained at approximately 30–60 min/week of muscle-strengthening activities, and the risk of diabetes sharply decreased until 60 min/week of muscle-strengthening activities, followed by a gradual decrease.
