# Supplementary material for: Muscle-strengthening activities are associated with lower risk and mortality in major non-communicable diseases: a systematic review and meta-analysis of cohort studies
Source: Br J Sports Med. 2022 Feb 28;56(13):755–63. doi: 10.1136/bjsports-2021-105061 (PMC9209691; doi:10.1136/bjsports-2021-105061)
Supplement: Supplementary data [file bjsports-2021-105061supp001.pdf]

Appendix Table 1. Search strategy for Ovid MEDLINE and Embase

Ovid MEDLINE

#	Searches
1	exp resistance training/
2	((resistance adj2 (exercise* OR training*)) OR (weight bearing adj2 (exercise* OR training* OR strengthening*)) OR (strength adj2 (exercise* OR training*)) OR (strengthening adj2 activit*) OR weightlifting OR (weight adj1 training) OR (weight adj1 lifting) OR ((muscular OR muscle) adj1 strengthening) OR (circuit adj1 training) OR (isometric adj1 exercise) OR (resistance exercise* OR resistance training OR weight bearing exercise* OR weight bearing training OR weight bearing strengthening OR strength exercise OR strength training OR strengthening activit* OR weight training OR weight lifting OR muscular strengthening OR muscle strengthening OR circuit training OR isometric exercise)).mp.
3	exp cohort studies/ OR (cohort OR prospective OR retrospective OR longitudinal OR (follow adj1 up) OR follow up).mp. OR observational study.pt. OR (exp health surveys/ OR health surve*.ti.)
4	mortality.mp. OR exp cause of death/ OR (death OR mortality).hw.
5	exp risk/ OR exp causality/ OR exp mortality/ OR (etiology or mortality).fs. OR exp morbidity/ OR (prevalen* OR morbidity or incidence).mp. OR (risk* OR mortality OR death OR cause* OR causality OR etiology OR incidence).ti.
6	(1 or 2) and 3 AND (4 OR 5)
7	((systematic adj1 review*) OR (meta adj1 analys*) OR (random* OR case report*) OR (phase adj1 (II OR III OR "2" OR "3")) OR phase II OR phase III OR phase 2 OR phase 3).ti. OR (clinical trial, all OR meta analysis OR systematic reviews OR case reports OR guideline OR review OR practice guideline OR comment OR letter ORor news).pt.
8	6 NOT 7
9	l/8 en=y
10	l/9 hu=y
11	exp muridae/ or (animals or animal).hw. or (in vitro or in vivo or mouse or mice or rat or rats).ti.
12	9 NOT 11
13	10 OR 12

EMBASE

#	Searches
L1	SEA RESISTANCE TRAINING+PFT,NT/CT
L2	SEA (RESISTANCE OR STRENGTH)(2A)(EXERCISE? OR TRAINING?) OR WEIGHT(W)BEARING(2A)(EXERCISE? OR TRAINING? OR STRENGTHENING?) OR STRENGTHENING(2A)ACTIVIT? OR WEIGHTLIFTING OR WEIGHT(1A)(TRAINING OR LIFTING) OR (MUSCULAR OR MUSCLE)(1A)STRENGTHENING OR CIRCUIT(1A)TRAINING OR ISOMETRIC(1A)EXERCIS
L3	SEA (COHORT ANALYSIS+PFT,NT OR OBSERVATIONAL STUDY+PFT,NT OR HEALTH SURVEY+PFT,NT)/CT OR COHORT OR PROSPECTIVE OR RETROSPECTIVE OR LONGITUDINAL OR FOLLOW(1A)UP OR HEALTH(W)SURVE?/TI
L4	SEA MORTALITY OR CAUSE OF DEATH+PFT,NT/CT E DEATH+KT/CT
L5	SEA (DEATH/CT OR "BH3 INTERACTING DOMAIN DEATH AGONIST PROTEIN"/CT OR "BCL 2 INTERACTING MEDIATOR OF CELL DEATH"/CT OR "BCL ASSOCIATED DEATH PROTEIN"/CT OR "BCL-ASSOCIATED DEATH PROTEIN"/CT OR "DISC (DEATH INDUCING SIGNALING COMPLEX)"/CT OR "DEATH ANXIETY SCALE"/CT OR "DEATH DEPRESSION SCALE"/CT OR "EDAR ASSOCIATED DEATH DOMAIN PROTEIN"/CT OR "EDAR-ASSOCIATED DEATH DOMAIN PROTEIN"/CT OR "FAS ASSOCIATED DEATH DOMAIN LIKE INTERLEUKIN 1BETA CONVERTING ENZYME"/CT OR "FAS ASSOCIATED DEATH DOMAIN LIKE INTERLEUKIN 1BETA CONVERTING ENZYME 2"/CT OR "FAS ASSOCIATED DEATH DOMAIN LIKE INTERLEUKIN 1BETA CONVERTING ENZYME INHIBITORY PROTEIN"/CT OR "FAS ASSOCIATED DEATH DOMAIN PROTEIN"/CT OR "FAS ASSOCIATED DEATH DOMAIN PROTEIN INTERLEUKIN1BETA CONVERTING ENZYME 2"/CT OR "FAS ASSOCIATING DEATH DOMAIN PROTEIN"/CT OR "FAS-ASSOCIATED DEATH DOMAIN PROTEIN"/CT OR "IUFD (INTRAUTERINE FETAL DEATH)"/CT OR "MN-INDUCED NEURONAL CELL DEATH"/CT OR "MN-INDUCED NEURONAL DEATH"/CT OR "PARP-1-DEPENDENT CELL DEATH"/CT OR "PARP-DEPENDENT CELL DEATH"/CT OR "PROGRAMMED DEATH-LIGAND 1 IMMUNOHISTOCHEMISTRY ASSAY"/CT OR "PROGRAMMED DEATH-LIGAND 1 TEST KIT"/CT OR "RIP ASSOCIATED PROTEIN WITH A DEATH DOMAIN"/CT OR "TNF RECEPTOR ASSOCIATED DEATH DOMAIN PROTEIN"/CT OR "TNF RECEPTOR-ASSOCIATED DEATH DOMAIN PROTEIN"/CT OR "TNF RELATED DEATH LIGAND 1"/CT OR "ACCIDENTAL DEATH"/CT OR "ACTIVATION INDUCED CELL DEATH"/CT OR "AFTER-DEATH CARE"/CT OR "ANTEPARTUM DEATH"/CT OR "APOPTOTIC CELL DEATH"/CT OR "APOPTOTIC CELLULAR DEATH"/CT OR "APOPTOTIC DEATH"/CT OR "APOPTOTIC NERVE CELL DEATH"/CT OR "APOPTOTIC NEURON DEATH"/CT OR "APOPTOTIC NEURONAL CELL DEATH"/CT OR "APOPTOTIC NEURONAL DEATH"/CT OR "APOPTOTIC-LIKE CELL DEATH"/CT OR "APOPTOTIC-LIKE NEURON DEATH"/CT OR "APOPTOTIC-LIKE NEURONAL CELL DEATH"/CT OR "APOPTOTIC-LIKE NEURONAL DEATH"/CT OR "APOPTOTIC-MEDIATED NEURONAL DEATH"/CT OR "ATTITUDE TO DEATH"/CT OR "AUTOPHAGIC CELL DEATH"/CT OR "AUTOPHAGIC PROGRAMMED CELL DEATH"/CT OR "AUTOPHAGY-DEPENDENT CELL DEATH"/CT OR "BATHTUB DEATH"/CT OR "BRAIN DEATH"<br>E MORTALITY+KT/CT
L6	SEA (MORTALITY/CT OR "100% MORTALITY TIME"/CT OR "50% MORTALITY LETHAL TIME"/CT OR "GRACE MORTALITY SCORE"/CT OR "GLOBAL REGISTRY OF ACUTE CORONARY EVENTS MORTALITY RISK SCORE"/CT OR "PAEDIATRIC INDEX OF MORTALITY"/CT OR "PAEDIATRIC INDEX OF MORTALITY 2"/CT OR "PEDIATRIC INDEX OF

	MORTALITY"/CT OR "PEDIATRIC INDEX OF MORTALITY 2"/CT OR "PEDIATRIC INDEX OF MORTALITY 2 SCORE"/CT OR "SMR (STANDARDISED MORTALITY RATIO)"/CT OR "SMR (STANDARDIZED MORTALITY RATIO)"/CT OR "SMRS (STANDARDISED MORTALITY RATIOS)"/CT OR "SMRS (STANDARDIZED MORTALITY RATIOS)"/CT OR "SOCIETY OF THORACIC SURGEONS MORTALITY RISK SCORE"/CT OR "ALL CAUSE MORTALITY"/CT OR "CANCER MORTALITY"/CT OR "CARDIOVASCULAR MORTALITY"/CT OR "CHILD MORTALITY"/CT OR "CHILDHOOD MORTALITY"/CT OR "DRUG MORTALITY"/CT OR "EMBRYO MORTALITY"/CT OR "EMBRYONIC MORTALITY"/CT OR "EXCESS MORTALITY"/CT OR "FETAL MORTALITY"/CT OR "FETUS MORTALITY"/CT OR "FOETAL MORTALITY"/CT OR "FOETUS MORTALITY"/CT OR "HOSPITAL MORTALITY"/CT OR "INFANT MORTALITY"/CT OR "INFANTILE MORTALITY"/CT OR "INTRAUTERINE MORTALITY"/CT OR "LETHAL TIME FOR 50% MORTALITY"/CT OR "LETHAL TIME OF MORTALITY"/CT OR "MATERNAL MORTALITY"/CT OR "MORTALITY CAUSE"/CT OR "MORTALITY MODEL"/CT OR "MORTALITY PREDICTION SCALE"/CT OR "MORTALITY PREDICTION SCORE"/CT OR "MORTALITY RATE"/CT OR "MORTALITY RISK"/CT OR "MORTALITY RISK INDEX"/CT OR "MORTALITY RISK SCALE"/CT OR "MORTALITY RISK SCORE"/CT OR "MORTALITY, CANCER"/CT OR "MORTALITY, CHILDHOOD"/CT OR "MORTALITY, FETAL"/CT OR "MORTALITY, FOETAL"/CT OR "MORTALITY, INFANT"/CT OR "MORTALITY, MATERNAL"/CT OR "MORTALITY, NEONATAL"/CT OR "MORTALITY, PERINATAL"/CT OR "MORTALITY, PREMATURE"/CT OR "MORTALITY, PRENATAL"/CT OR "MOTHER MORTALITY"/CT OR "NEONATAL MORTALITY"/CT OR "NEONATUS MORTALITY"/CT OR "NEWBORN MORTALITY"/CT OR "OPERATION MORTALITY"/CT OR "OPERATIVE MORTALITY"/CT OR "PERINATAL MORTALITY"/CT OR "POSTNEONATAL MORTALITY"/CT OR "POSTOPERATIVE MORTALITY"/CT OR "POULTENTERITIS MORTALITY SYNDROME"/CT OR "PREMATURE MORTALITY"/CT OR "PRENATAL MORTALITY"/CT OR "
L7	QUE (RISK+PFT,NT OR CAUSALITY+PFT,NT OR MORTALITY+PFT,NT OR MORBIDITY+PFT,NT)/CT OR ET/CT OR PREVALEN? OR MORBIDITY OR INCIDENCE OR (RISK? OR MORTALITY OR DEATH OR CAUSE? OR CAUSALITY OR ETIOLOGY OR INCIDENCE)/TI
L8	SEA (L1 OR L2) AND L3 AND (L4 OR L5 OR L6 OR L7)
L9	SEA (SYSTEMATIC(1A)REVIEW? OR META(1A)ANALYS? OR RANDOM? OR CASE(W)REPORT? OR PHASE(1A)(II OR III OR 2 OR 3))/TI OR (LETTER OR REVIEW)/DT
L10	QUE (CLINICAL TRIAL+PFT,NT OR META ANALYSIS+PFT,NT OR SYSTEMATIC REVIEW+PFT,NT OR CASE REPORT+PFT,NT OR REVIEW+PFT,NT OR PRACTICE GUIDELINE+PFT,NT OR LETTER+PFT,NT)/CT
L11	SEA L8 NOT (L9 OR L10)
L12	SEA L11 AND ENGLISH/LA
L13	SEA L12 AND HUMAN+AUTO/CT
L14	QUE MURIDAE+PFT,NT/CT OR (IN(W)VITRO OR IN(W)VIVO OR MOUSE OR MICE OR RAT OR RATS)/TI<br>E ANIMALS+KT/CT
L15	SEA (ANIMALS/CT OR "ADULT ANIMALS"/CT OR "ANIMALS BY OUTER APPEARANCE"/CT OR "ANIMALS, CONGENIC"/CT OR "ANIMALS, DOMESTIC"/CT OR "ANIMALS, EXOTIC"/CT OR "ANIMALS, GENETICALLY MODIFIED"/CT OR "ANIMALS, INBRED STRAINS"/CT OR "ANIMALS, LABORATORY"/CT OR "ANIMALS, NEWBORN"/CT OR "ANIMALS, OUTBRED STRAINS"/CT OR "ANIMALS, POISONOUS"/CT OR "ANIMALS, SUCKLING"/CT OR "ANIMALS, TRANSGENIC"/CT OR "ANIMALS, WILD"/CT OR "ANIMALS, ZOO"/CT OR "BULLOCKS (DRAFT

	ANIMALS)"/CT OR "CANINES (ANIMALS)"/CT OR "CONGENIC ANIMALS"/CT OR "CONTAMINATED ANIMALS"/CT OR "CRUELTY TO ANIMALS"/CT OR "DAMS (ANIMALS)"/CT OR "DOMESTIC ANIMALS"/CT OR "DOMESTICATED ANIMALS"/CT OR "EXOTIC ANIMALS"/CT OR "EXPERIMENTAL ANIMALS"/CT OR "FARM ANIMALS"/CT OR "FERAL ANIMALS"/CT OR "FOSSIL ANIMALS"/CT OR "GENETICALLY MODIFIED ANIMALS"/CT OR "ILLEGAL TRADE IN WILD ANIMALS"/CT OR "ILLEGAL TRADE OF WILD ANIMALS"/CT OR "ILLEGAL TRADING OF WILD ANIMALS"/CT OR "INBRED STRAINS ANIMALS"/CT OR "LABORATORY ANIMALS"/CT OR "MATURE ANIMALS"/CT OR "MYTHICAL ANIMALS"/CT OR "NON-NATIVE ANIMALS"/CT OR "OUTBRED STRAINS ANIMALS"/CT OR "PET ANIMALS"/CT OR "POISONOUS ANIMALS"/CT OR "POLLINATION BY ANIMALS"/CT OR "POLLUTED ANIMALS"/CT OR "SEX WITH ANIMALS"/CT OR "SUCKLING ANIMALS"/CT OR "TOXIC ANIMALS"/CT OR "TRANSGENIC ANIMALS"/CT OR "VENOMOUS ANIMALS"/CT OR "WILD ANIMALS"/CT OR "WORKING ANIMALS"/CT OR "ZOO ANIMALS"/CT)<br>E ANIMAL+KT/CT
L16	QUE (ANIMAL/CT OR "AFRICAN ANIMAL TRYPANOSOMIASIS"/CT OR "AFRICAN ANIMAL TRYPANOSOMOSIS"/CT OR "DIO ANIMAL"/CT OR "MEAT ANIMAL RESEARCH CENTER-145 (MARC-145) CELL LINE"/CT OR "MEAT ANIMAL RESEARCH CENTER-145 (CELL LINE)"/CT OR "PHOLIDOTA (ANIMAL)"/CT OR "ABNORMAL ANIMAL BEHAVIOR"/CT OR "ABNORMAL BEHAVIOR (ANIMAL)"/CT OR "ADULT ANIMAL"/CT OR "ALPHA ANIMAL"/CT OR "ALTRICIAL ANIMAL"/CT OR "ANATOMY, ANIMAL"/CT OR "ANIMAL AFRICAN TRYPANOSOMIASIS"/CT OR "ANIMAL ABNORMAL BEHAVIOR"/CT OR "ANIMAL ABNORMAL BEHAVIOUR"/CT OR "ANIMAL ABUSE"/CT OR "ANIMAL ANAESTHESIA"/CT OR "ANIMAL ANATOMY"/CT OR "ANIMAL ANESTHESIA"/CT OR "ANIMAL ASSISTED THERAPY"/CT OR "ANIMAL BEHAVIOR"/CT OR "ANIMAL BEHAVIOR PROBLEM"/CT OR "ANIMAL BEHAVIORAL PROBLEM"/CT OR "ANIMAL BEHAVIOUR"/CT OR "ANIMAL BEHAVIOUR PROBLEM"/CT OR "ANIMAL BITE"/CT OR "ANIMAL BREEDING"/CT OR "ANIMAL CAGE"/CT OR "ANIMAL CARE"/CT OR "ANIMAL CARE COMMITTEES"/CT OR "ANIMAL CARE HOSPITAL"/CT OR "ANIMAL CARE TECHNICIAN"/CT OR "ANIMAL CARE TECHNICIANS"/CT OR "ANIMAL CASTE"/CT OR "ANIMAL CELL"/CT OR "ANIMAL CELL CULTURE"/CT OR "ANIMAL COLONY"/CT OR "ANIMAL COMFORT"/CT OR "ANIMAL COMMUNICATION"/CT OR "ANIMAL COMMUNITIES"/CT OR "ANIMAL COMMUNITY"/CT OR "ANIMAL CONIINE"/CT OR "ANIMAL CRUELTY"/CT OR "ANIMAL CULLING"/CT OR "ANIMAL DERIVED BIOABSORBABLE BONE MATRIX IMPLANT"/CT OR "ANIMAL DERIVED BONE MATRIX IMPLANT"/CT OR "ANIMAL DISEASE"/CT OR "ANIMAL DISEASE MODEL"/CT OR "ANIMAL DISEASE MODELS"/CT OR "ANIMAL DISEASES"/CT OR "ANIMAL DISPERSAL"/CT OR "ANIMAL DISTRIBUTION"/CT OR "ANIMAL DOCTOR"/CT OR "ANIMAL EMBRYO"/CT OR "ANIMAL EUTHANASIA"/CT OR "ANIMAL EXPERIMENT"/CT OR "ANIMAL EXPERIMENTATION"/CT OR "ANIMAL EXTRACT"/CT OR "ANIMAL EXTRACTS"/CT OR "ANIMAL FACILITATED THERAPY"/CT OR "ANIMAL FEED"/CT OR "ANIMAL FIBER"/CT OR "ANIMAL FIBRE"/CT OR "ANIMAL FIN"/CT OR "ANIMAL FINS"/CT OR "ANIMAL FLIGHT"/CT OR "ANIMAL FOOD"/CT OR "ANIMAL FUR"/CT OR "ANIMAL GENETICS"/CT OR "ANIMAL HEALTH"/CT OR "ANIMAL HEALTH AIDES"/CT OR "ANIMAL HEALTH ASSISTANT"/CT OR "ANIMAL HEALTH TECHNICIAN"/CT OR "
L17	SEA L12 NOT (L14 OR L15 OR L16)
L18	SEA L13 OR L17
L19	SEA L18 NOT CONFERENC?/DT

	ACT HTITLE/Q
L20-188	We entered the information about the records identified through MEDLINE searching and removed the overlaps with MEDLINE

Appendix Table 2. Quality assessment of included studies according to Newcastle-Ottawa Scale

	Newcastle-Ottawa Scale items for cohort studies								
	**Selection**			**Comparability**		**Outcome**			**Stars**
First author, year	Selection of the non-exposed cohort	Ascertainment of exposure	Demonstration that outcome was not present at start of study	Multivariate adjustment	Aerobic physical activity	Assessment of outcome	Length of follow-up	Adequacy of follow-up	
**All-cause mortality**									
Grøntved, 2012[19]	A*	C	A*	A*	A*	A*	A*	B*	7
Kamada, 2017[42]	A*	C	A*	A*	A*	A*	A*	A*	7
Stamatakis, 2018[20]	A*	C	A*	A*	A*	A*	A*	D	6
Liu, 2019[44]	A*	C	A*	A*	A*	A*	A*	C	6
Sheehan, 2020[18]	A*	B*	B	A*	A*	A*	A*	B*	7
Porter, 2020[49]	A*	B*	B	A*	A*	A*	A*	A*	7
Patel, 2020[48]	A*	C	A*	A*	A*	A*	A*	D	6
Zhao, 2020[51]	A*	B*	B	A*	-	A*	A*	D	5
**CVD**									
Grøntved, 2012[19]	A*	C	A*	A*	A*	A*	A*	B*	7
Kamada, 2017[42]	A*	C	A*	A*	A*	A*	A*	A*	7
Shiroma, 2017[43]	A*	C	A*	A*	A*	C	A*	A*	6
Stamatakis, 2018[20]	A*	C	A*	A*	A*	B*	A*	D	6
Liu, 2019[44]	A*	C	A*	A*	A*	A*	A*	C	6
Porter, 2019[46]	A*	B*	A*	A*	A*	B*	A*	D	7
Porter, 2020[49]	A*	B*	B	A*	A*	B*	A*	A*	7
Patel, 2020[48]	A*	C	A*	A*	A*	B*	A*	D	6
Zhao, 2020[51]	A*	B*	B	A*	-	A*	A*	D	5

**Total cancer/site-specific cancers incidence**									
Kamada, 2017[42]	A*	C	A*	A*	A*	A*	A*	A*	7
Stamatakis, 2018[20]	A*	C	A*	A*	A*	A*	A*	D	6
Siahpush, 2019[47]	A*	B*	B	A*	A*	A*	A*	B*	7
Mazzilli, 2019[45]	A*	C	A*	A*	A*	A*	A*	D	6
Porter, 2020[49]	A*	B*	B	A*	A*	A*	A*	A*	7
Patel, 2020[48]	A*	C	A*	A*	A*	A*	A*	D	6
Rezende, 2020[50]	A*	C	A*	A*	A*	A*	A*	B*	7
Zhao, 2020[51]	A*	B*	B	A*	-	A*	A*	D	5
**Diabetes incidence**									
Grøntved, 2012[19]	A*	C	A*	A*	A*	B	A*	B*	6
Grøntved, 2014[40]	A*	C	A*	A*	A*	B	A*	B*	6
Kuwahara, 2015[41]	A*	C	A*	B	A*	A*	A*	B*	6
Shiroma, 2017[43]	A*	C	A*	A*	A*	B	A*	A*	6
Mielke, 2020[17]	A*	C	A*	B	A*	B	A*	C	4

Quality Assessment

The quality of the studies was assessed using a modification of the Newcastle-Ottawa Scale (NOS) for quality assessment of prospective cohort studies.[22] We excluded the "representativeness of the exposed cohort" item of the original NOS because our quality assessment was planned to evaluate internal validity, not external validity. Therefore, 8 stars in total were achievable. HM and RK independently assessed the studies and resolved any inconsistencies through a discussion.

Criteria of quality assessment

1. Selection of the nonexposed cohort

A: Participants with and without muscle-strengthening activities were selected from the same source population. (*)
B: Participants with and without muscle-strengthening activities were not selected from the same source population.
C: No description.

2. Ascertainment of exposure

A: An objective method was used to assess muscle-strengthening activities. (*)
B: A structured interview was used to assess muscle-strengthening activities. (*)

C: A self-reported questionnaire was used to assess muscle-strengthening activities.
D: No description.

3. Demonstration that the outcome of interest was not present at the start of the study
A: Exclusion of participants with baseline cardiovascular diseases (both stroke or coronary heart disease) and/or cancer in analyses of all-cause mortality, participants with baseline cardiovascular diseases in analyses of cardiovascular disease mortality, participants with cancer in analyses of cancer mortality, and participants with baseline outcomes of interest in analyses of incidence of noncommunicable diseases. (*)
B: No exclusion of participants with the abovementioned outcomes.
C: No description.

4. Comparability of cohorts on the basis of the design or analysis
(1) Multivariate adjustment
A: The study adjusted for at least three of five covariates (smoking, alcohol consumption, diet, body composition, socioeconomic status) in addition to age, sex, and race/ethnicity, if relevant. (*)
B: The study did not adjust for these covariates.
(2) Aerobic physical activity
A: The study adjusted for aerobic physical activity. (*)
B: The study did not adjust for aerobic physical activity.

5. Assessment of outcome
A: Patient registers or death certificates for mortality and clinical assessment, medical records, or record linkage for incidence. (*)
B: Self-report.
C: No description.

6. Length of follow-up
A: The follow-up period was a mean/median of ≥5 years. (*)
B: The follow-up period was <5 years.

7. Adequacy of follow-up of cohorts
A: Participants were completely (≥99%) followed up. (*)
B: Approximately ≥80% of the participants were followed up or the description of participants lost to follow-up indicated that bias was unlikely to have been introduced. (*)
C: Less than 80% of the participants were followed up.
D: No description.

Reference
22 Wells GA, Shea B, O'Connell D, et al. The Newcastle-Ottawa Scale (NOS) for assessing the quality of nonrandomised studies in meta-analyses. 2009. Available from: http://www.ohri.ca/programs/clinical_epidemiology/oxford.asp accessed January 5th, 2021.

Appendix Table 3. GRADE evidence profiles for the association of muscle-strengthening activities and the risk of mortality and noncommunicable disease

Grading the evidence

The Grading of Recommendations, Assessment, Development and Evaluation (GRADE) approach was used to assess the overall certainty of evidence for outcomes with results from two or more studies.[33] GRADE assesses the evidence as very low, low, moderate, or high quality. One reviewer (HM) assessed the certainty of evidence, whereas two reviewers (RK and TH) examined and revised the certainty of assessments, as necessary. The certainty of evidence starts at a low level because of the inherent limitations of observational studies. The downgraded criteria included risk of bias (weight of studies showing a risk of bias according to a low NOS [<6]), inconsistency (similarity of point estimates, extent of overlap of confidence intervals [CIs], same direction of effects, $I^2 \geq 50\%$, and $p<0.10$),[34] indirectness (presence of factors that limit the generalizability of the results),[35] imprecision,[36] and publication bias.[37] On the basis of the literature,[36] we considered the optimal information size to be 400 cases and 4000 participants with a 25% relative risk (RR) reduction. If the optimal information size criterion was not met, the evidence was downgraded for imprecision.[36] We also downgraded for imprecision when the optimal information size criterion was met but the 95% CI included 1.00 and the upper and lower bounds of 95% CI were <0.75 and >1.25, respectively.[36] The upgraded criteria included a large magnitude of effect (RR>2 or RR<0.5 in the absence of plausible confounders), dose-response gradient, or opposing residual confounding.[38] A GRADE evidence profile was developed.[39]

Certainty assessment								Summary of findings			Certainty
								No. of participants		Effect	
No. of studies	Study design	Risk of bias	Inconsistency	Indirectness	Imprecision	Publication bias	Other considerations	Participants	Cases	Relative (95% CI)	
**All-cause mortality**											
7	Observational studies	Not serious	Not serious[a]	Serious[b]	Not serious	NA[c]	NA	263 058	42 133	0.85 (0.79 to 0.93)	⊕◯◯◯ VERY LOW
**Cardiovascular diseases**											
7	Observational studies	Not serious	Not serious[a]	Serious[b]	Not serious	NA[c]	NA	257 888	16 056	0.83 (0.73 to 0.93)	⊕◯◯◯ VERY LOW
**Total cancer**											
6	Observational studies	Not serious	Not serious[a]	Serious[b]	Not serious	NA[c]	NA	540 543	21 253	0.88 (0.80 to 0.97)	⊕◯◯◯ VERY LOW
**Diabetes incidence**											
5	Observational studies	Not serious	Not serious	Not serious	Not serious	NA[c]	Dose-response gradient[d]	202 486	9548	0.83 (0.77 to 0.89)	⊕⊕◯◯ LOW
**Colon cancer incidence**											

Certainty assessment								Summary of findings			Certainty
								No. of participants		Effect	
No. of studies	Study design	Risk of bias	Inconsistency	Indirectness	Imprecision	Publication bias	Other considerations	Participants	Cases	Relative (95% CI)	
2	Observational studies	Not serious	Not serious	Serious[b]	Not serious	NA[c]	NA	248 909	2415	0.96 (0.91 to 1.01)	⊕○○○ VERY LOW
**Kidney cancer incidence**											
2	Observational studies	Not serious	Not serious	Serious[b]	Serious[e]	NA[c]	NA	248 909	1063	0.88 (0.76 to 1.02)	⊕○○○ VERY LOW
**Bladder cancer incidence**											
2	Observational studies	Not serious	Not serious	Serious[b]	Not serious	NA[c]	NA	248 909	2341	0.94 (0.84 to 1.05)	⊕○○○ VERY LOW
**Lung cancer incidence**											
2	Observational studies	Not serious	Not serious	Serious[b]	Not serious	NA[c]	Dose-response gradient[n]	248 909	4075	0.90 (0.83 to 0.98)	⊕○○○ VERY LOW
**Pancreatic cancer incidence**											
2	Observational studies	Not serious	Not serious	Serious[b]	Serious[e]	NA[c]	NA	248 909	1028	1.12 (0.98 to 1.28)	⊕○○○ VERY LOW

CI: Confidence interval

[a] Despite the high I^2 and p<0.10 judged as not serious because of the overlapping CI and same direction of effects in the forest plots

[b] Downgraded by one level because all studies were conducted in Western countries, especially in USA

[c] Publication bias could not be assessed due to limited number of studies

[d] Not upgraded despite the dose-response gradient because publication bias could not be assessed

[e] Serious imprecision because optimal information size was not met

[f] Serious imprecision because the 95% CI include the null value (1.00) and the upper bound=1.25, although optimal information size met (cases=1028, participants=248 909)

Reference

33 Guyatt G, Oxman AD, Akl EA, et al. GRADE guidelines: 1. Introduction-GRADE evidence profiles and summary of findings tables. J Clin Epidemiol 2011;64(4):383-94. doi: 10.1016/j.jclinepi.2010.04.026

34 Guyatt GH, Oxman AD, Kunz R, et al. GRADE guidelines: 7. Rating the quality of evidence--inconsistency. J Clin Epidemiol 2011;64(12):1294-302. doi: 10.1016/j.jclinepi.2011.03.017

35 Guyatt GH, Oxman AD, Kunz R, et al. GRADE guidelines: 8. Rating the quality of evidence--indirectness. J Clin Epidemiol 2011;64(12):1303-10. doi: 10.1016/j.jclinepi.2011.04.014

36 Guyatt GH, Oxman AD, Kunz R, et al. GRADE guidelines 6. Rating the quality of evidence--imprecision. J Clin Epidemiol 2011;64(12):1283-93. doi: 10.1016/j.jclinepi.2011.01.012

37 Guyatt GH, Oxman AD, Montori V, et al. GRADE guidelines: 5. Rating the quality of evidence--publication bias. J Clin Epidemiol 2011;64(12):1277-82. doi: 10.1016/j.jclinepi.2011.01.011

38 Guyatt GH, Oxman AD, Sultan S, et al. GRADE guidelines: 9. Rating up the quality of evidence. J Clin Epidemiol 2011;64(12):1311-6. doi: 10.1016/j.jclinepi.2011.06.004

39 Guyatt GH, Oxman AD, Santesso N, et al. GRADE guidelines: 12. Preparing summary of findings tables-binary outcomes. J Clin Epidemiol 2013;66(2):158-72. doi: 10.1016/j.jclinepi.2012.01.012

Appendix Table 4. Outcomes excluded from our meta-analysis

First author, year	Country; Cohort	Participants characteristics	Outcome; Case ascertainment; Case/Participants	Follow-up	Exposure measurement	Exposure category	Effect estimates	Covariates	Quality assessment
**Chronic lower respiratory tract diseases mortality**									
Zhao, 2020[51]	USA; NHIS	Men and women; ≥18 years	Chronic lower respiratory tract diseases mortality; NDI; 3188/479 856	8.75 years (median)	Interview	Neither guideline<br>Aerobic only<br>Strength only<br>Both guideline	1<br>0.42 (0.37 to 0.47)<br>0.76 (0.62 to 0.93)<br>0.29 (0.23 to 0.37)	Sex, age, race/ethnicity, education, marital status, BMI, smoking status, alcohol intake, and chronic conditions (hypertension, heart disease, stroke, cancer, and diabetes)	5
**Accidents and injuries mortality**									
Zhao, 2020[51]	USA; NHIS	Men and women; ≥18 years	Accidents and injuries mortality; NDI; 2477/479 856	8.75 years (median)	Interview	Neither guideline<br>Aerobic only<br>Strength only<br>Both guideline	1<br>0.82 (0.73 to 0.93)<br>1.08 (0.87 to 1.35)<br>0.71 (0.60 to 0.84)	Sex, age, race/ethnicity, education, marital status, BMI, smoking status, alcohol intake, and chronic conditions (hypertension, heart disease, stroke, cancer, and diabetes)	5
**Alzheimer's disease mortality**									
Zhao, 2020[51]	USA; NHIS	Men and women; ≥18 years	Alzheimer's disease mortality; NDI; 1470/479 856	8.75 years (median)	Interview	Neither guideline<br>Aerobic only<br>Strength only<br>Both guideline	1<br>0.74 (0.62 to 0.87)<br>0.88 (0.64 to 1.23)<br>0.64 (0.48 to 0.86)	Sex, age, race/ethnicity, education, marital status, BMI, smoking status, alcohol intake, and chronic conditions (hypertension, heart disease, stroke, cancer, and diabetes)	5
**Diabetes mellitus mortality**									
Zhao, 2020[51]	USA; NHIS	Men and women; ≥18 years	Diabetes mellitus mortality; NDI; 1803/479 856	8.75 years (median)	Interview	Neither guideline<br>Aerobic only<br>Strength only<br>Both guideline	1<br>0.63 (0.53 to 0.74)<br>0.96 (0.73 to 1.27)<br>0.47 (0.36 to 0.62)	Sex, age, race/ethnicity, education, marital status, BMI, smoking status, alcohol intake, and chronic conditions (hypertension, heart disease, stroke, cancer, and diabetes)	5
**Influenza and pneumonia mortality**									
Zhao, 2020[51]	USA; NHIS	Men and women; ≥18 years	Influenza and pneumonia mortality; NDI; 1135/479 856	8.75 years (median)	Interview	Neither guideline<br>Aerobic only<br>Strength only<br>Both guideline	1<br>0.55 (0.44 to 0.68)<br>0.87 (0.62 to 1.24)<br>0.46 (0.32 to 0.64)	Sex, age, race/ethnicity, education, marital status, BMI, smoking status, alcohol intake, and chronic conditions (hypertension, heart disease, stroke, cancer, and diabetes)	5

First author, year	Country; Cohort	Participants characteristics	Outcome; Case ascertainment; Case/Participants	Follow-up	Exposure measurement	Exposure category	Effect estimates	Covariates	Quality assessment
**Nephritis, nephrotic syndrome, or nephrosis mortality**									
Zhao, 2020[51]	USA; NHIS	Men and women; ≥18 years	Nephritis, nephrotic syndrome, or nephrosis mortality; NDI; 1129/479 856	8.75 years (median)	Interview	Neither guideline<br>Aerobic only<br>Strength only<br>Both guideline	1<br>0.48 (0.40 to 0.59<br>0.71 (0.50 to 1.01)<br>0.52 (0.36 to 0.76)	Sex, age, race/ethnicity, education, marital status, BMI, smoking status, alcohol intake, and chronic conditions (hypertension, heart disease, stroke, cancer, and diabetes)	5
**Other cause-specific mortality**									
Hsu, 2018[21]	Australia; CHAMP	Men; ≥70 years (mean 77 years)	Cancer mortality; New South Wales Registry of Births, Deaths, and Marriages; -/958	7 years (median)	Questionnaire	No<br>Yes	1<br>-	Age, comorbidity, smoking status, alcohol, BMI, ethnicity, education, diabetes, health-related quality of life, activities of daily living disability, depression, and PASE score	5
**Breast cancer incidence**									
Mazzilli, 2019[45]	USA, NIH-AARP DHS	Men and women; 50-71 years	Breast cancer; Cancer registries; 3288/215 122	10 years (max)	Questionnaire	None<br>5-90 min/week<br>≥120 min/week	1<br>1.02 (0.93 to 1.11)<br>0.99 (0.83 to 1.17)	Age, sex, BMI, smoking status, race, education, alcohol intake, MVPA not including weight lifting, oral birth control use, age of menarche, age of menopause, postmenopausal hormone use, and parity	6
**Lymphoma incidence**									
Mazzilli, 2019[45]	USA, NIH-AARP DHS	Men and women; 50-71 years	Non-Hodgkin's lymphoma; Cancer registries; 1187/215 122	10 years (max)	Questionnaire	None<br>5-90 min/week<br>≥120 min/week	1<br>0.90 (0.78 to 1.05)<br>0.96 (0.75 to 1.23)	Age, sex, BMI, smoking status, race, education, alcohol intake, and MVPA not including weight lifting	6

First author, year	Country; Cohort	Participants characteristics	Outcome; Case ascertainment; Case/Participants	Follow-up	Exposure measurement	Exposure category	Effect estimates	Covariates	Quality assessment
Rezende, 2020[50]	USA, HPSF	Men; 40-75 years (mean 67.5 years)	Lymphoma; Self-reported cancer diagnosis confirmed from medical records or NDI; 484/33 787	24 years (max)	Questionnaire	None<br>1-59 min/week<br>≥60 min/week	1<br>1.02 (0.81 to 1.29)<br>1.08 (0.79 to 1.50)	Race, height, family history of cancer, physical exam in past two years, history of colonoscopy or sigmoidoscopy, smoking in pack years, regular aspirin use, multivitamin use, alcohol consumption, red and processed meat intake, Alternate Healthy Eating Index, prostate-specific antigen test in past 2 years, total physical activity except for resistance training, total energy intake, and BMI	7
**Prostate cancer incidence**									
Mazzilli, 2019[45]	USA, NIH-AARP DHS	Men and women; 50-71 years	Prostate cancer; Cancer registries; 7213/215 122	10 years (max)	Questionnaire	None<br>5-90 min/week<br>≥120 min/week	1<br>1.03 (0.97 to 1.09)<br>1.05 (0.96 to 1.15)	Age, sex, BMI, smoking status, race, education, alcohol intake, and MVPA not including weight lifting	6
Rezende, 2020[50]	USA, HPSF	Men; 40-75 years (mean 67.5 years)	Advanced prostate cancer; Self-reported cancer diagnosis confirmed from medical records or NDI; 657/33 787	24 years (max)	Questionnaire	None<br>1-59 min/week<br>≥60 min/week	1<br>0.96 (0.77 to 1.20)<br>0.89 (0.66 to 1.19)	Race, height, family history of cancer, physical exam in past two years, history of colonoscopy or sigmoidoscopy, smoking in pack years, regular aspirin use, multivitamin use, alcohol consumption, red and processed meat intake, Alternate Healthy Eating Index, prostate-specific antigen test in past 2 years, total physical activity except for resistance training, total energy intake, and BMI	7
**Rectum cancer incidence**									
Mazzilli, 2019[45]	USA, NIH-AARP DHS	Men and women; 50-71 years	Rectum cancer; Cancer registries; 527/215 122	10 years (max)	Questionnaire	None<br>5-90 min/week<br>≥120 min/week	1<br>0.68 (0.52 to 0.88)<br>1.01 (0.69 to 1.48)	Age, sex, BMI, smoking status, race, education, alcohol intake, and MVPA not including weight lifting	6
**Melanoma incidence**									

First author, year	Country; Cohort	Participants characteristics	Outcome; Case ascertainment; Case/Participants	Follow-up	Exposure measurement	Exposure category	Effect estimates	Covariates	Quality assessment
Mazzilli, 2019[45]	USA, NIH-AARP DHS	Men and women; 50-71 years	Melanoma; Cancer registries; 2454/215 122	10 years (max)	Questionnaire	None<br>5-90 min/week<br>≥120 min/week	1<br>1.18 (1.07 to 1.30)<br>1.03 (0.88 to 1.20)	Age, sex, BMI, smoking status, race, education, alcohol intake, and MVPA not including weight lifting	6
**Leukemia incidence**									
Rezende, 2020[50]	USA, HPSF	Men; 40-75 years (mean 67.5 years)	Leukemia; Self-reported cancer diagnosis confirmed from medical records or NDI; 188/33 787	24 years (max)	Questionnaire	None<br>1-59 min/week<br>≥60 min/week	1<br>0.81 (0.55 to 1.19)<br>1.00 (0.59 to 1.70)	Race, height, family history of cancer, physical exam in past two years, history of colonoscopy or sigmoidoscopy, smoking in pack years, regular aspirin use, multivitamin use, alcohol consumption, red and processed meat intake, Alternate Healthy Eating Index, prostate-specific antigen test in past 2 years, total physical activity except for resistance training, total energy intake, and BMI	7
**Multiple myeloma incidence**									
Rezende, 2020[50]	USA, HPSF	Men; 40-75 years (mean 67.5 years)	Multiple myeloma; Self-reported cancer diagnosis confirmed from medical records or NDI; 112/33 787	24 years (max)	Questionnaire	None<br>1-59 min/week<br>≥60 min/week	1<br>0.99 (0.61 to 1.60)<br>0.93 (0.46 to 1.89)	Race, height, family history of cancer, physical exam in past two years, history of colonoscopy or sigmoidoscopy, smoking in pack years, regular aspirin use, multivitamin use, alcohol consumption, red and processed meat intake, Alternate Healthy Eating Index, prostate-specific antigen test in past 2 years, total physical activity except for resistance training, total energy intake, and BMI	7
**Oesophageal cancer incidence**									

First author, year	Country; Cohort	Participants characteristics	Outcome; Case ascertainment; Case/Participants	Follow-up	Exposure measurement	Exposure category	Effect estimates	Covariates	Quality assessment
Rezende, 2020[50]	USA, HPSF	Men; 40-75 years (mean 67.5 years)	Oesophageal cancer; Self-reported cancer diagnosis confirmed from medical records or NDI; 103/33787	24 years (max)	Questionnaire	None<br>1-59 min/week<br>≥60 min/week	1<br>1.27 (0.77 to 2.09)<br>0.71 (0.30 to 1.72)	Race, height, family history of cancer, physical exam in past two years, history of colonoscopy or sigmoidoscopy, smoking in pack years, regular aspirin use, multivitamin use, alcohol consumption, red and processed meat intake, Alternate Healthy Eating Index, prostate-specific antigen test in past 2 years, total physical activity except for resistance training, total energy intake, and BMI	7
**Ovarian cancer**									
Buras, 2021[62]	USA, NHS and NHSII	Women; NHS: 30-50 years (mean 65.8 years), NHSII: 25-42 years (mean 46.4 years)	Ovarian cancer. Self-report cancer diagnosis or linkage to the NDI confirmed from review of medical records, including pathology reports, or linkage to the relevant cancer registry; 609/109 294	-	Questionnaire	0 min/week<br>1-59 min/week<br>≥60 min/week	1<br>1.14 (0.93 to 1.39)<br>0.95 (0.74 to 1.22)	age, calendar year, cohort (NHS and NHSII), BMI, oral contraceptive use, parity, family history of breast or ovarian cancer, menopausal status, smoking, hormone therapy use, tubal ligation, hysterectomy, and other physical activity	6
**Hypertension incidence**									
Mielke, 2020[17]	Australia; HABITAT	Men and women; 40-65 years	Hypertension; Self-reported hypertension diagnosis; 1028/8784	6 years (max)	Questionnaire	None<br><1 time/week<br>≥1 time/week	1<br>0.89 (0.75 to 1.05)<br>0.82 (0.70 to 0.97)	Sex, age, education, annual income, living arrangements, cigarette smoking status, physical activity, diabetes, and obesity	4
**Hypercholesteremia incidence**									

First author, year	Country; Cohort	Participants characteristics	Outcome; Case ascertainment; Case/Participants	Follow-up	Exposure measurement	Exposure category	Effect estimates	Covariates	Quality assessment
Bakker, 2018[61]	USA; ACLS	Men and Women;<br>18-83 years<br>(mean 43 years)	Hypercholesteremia (NCEP-ATPIII);<br>Clinical assessment;<br>1430/7317	4 years (median)	Questionnaire	No<br>Yes	1<br>0.86 (0.76 to 0.98)	Age, examination year, BMI, current smoking, heavy alcohol drinking, abnormalities on electrocardiography, systolic and diastolic blood pressure, parental history of hypercholesterolemia, and aerobic exercise*<br>*Excluded from the joint analysis.	5
						0 min/week<br>1-59 min/week<br>60-119 min/week<br>120-179 min/week<br>≥180 min/week	1<br>0.68 (0.54 to 0.86)<br>0.93 (0.78 to 1.12)<br>0.86 (0.67 to 1.11)<br>0.98 (0.77 to 1.24)		
						0 time/week<br>1 time/week<br>2 times/week<br>3 times/week<br>4 times/week<br>≥5 times/week	1<br>0.77 (0.49 to 1.20)<br>0.69 (0.54 to 0.88)<br>0.93 (0.79 to 1.10)<br>0.84 (0.63 to 1.12)<br>1.02 (0.74 to 1.39)		
						Aerobic exercise (<500 MET• min/week) & Resistance training (<2 days/week)<br>Aerobic exercise (≥500 MET-min/week) & Resistance training (<2 days/week)<br>Aerobic exercise (<500 MET-min/week) & Resistance training (≥2 days/week)<br>Aerobic exercise (≥500 MET-min/week) & Resistance training (≥2 days/week)	1<br>0.89 (0.79 to 1.01)<br>0.82 (0.62 to 1.09)<br>0.79 (0.68 to 0.91)		
**Metabolic syndrome incidence**									

First author, year	Country; Cohort	Participants characteristics	Outcome; Case ascertainment; Case/Participants	Follow-up	Exposure measurement	Exposure category	Effect estimates	Covariates	Quality assessment
Bakker, 2017[60]	USA; ACLS	Men and women; (mean 46 years)	Metabolic syndrome (NCEP-ATPIII); Clinical assessment; 1147/7418	4 years (median)	Questionnaire	No<br>Yes<br><br><br>0 min/week<br>1-59 min/week<br>60-119 min/week<br>120-179 min/week<br>≥180 min/week<br><br><br>0 time/week<br>1 time/week<br>2 times/week<br>3 times/week<br>4 times/week<br>≥5 times/week<br><br><br>Aerobic exercise (<500 MET-min/week) & Resistance training (<2 days/week)<br>Aerobic exercise (≥500 MET-min/week) & Resistance training (<2 days/week)<br>Aerobic exercise (<500 MET-min/week) & Resistance training (≥2 days/week)<br>Aerobic exercise (≥500 MET-min/week) & Resistance training (≥2 days/week)	1<br>0.83 (0.72 to 0.95)<br><br><br>1<br>0.71 (0.56 to 0.89)<br>0.96 (0.80 to 1.16)<br>0.81 (0.61 to 1.07)<br>0.78 (0.60 to 1.02)<br><br><br>1<br>0.83 (0.54 to 1.27)<br>0.84 (0.67 to 1.06)<br>0.88 (0.74 to 1.05)<br>0.62 (0.44 to 0.89)<br>0.81 (0.56 to 1.16)<br><br><br>1<br>0.93<br>0.87<br>0.75 (0.63 to 0.89)	Age, sex, examination year, BMI, current smoking, heavy alcohol drinking, abnormal electrocardiographic findings, parental history of cardiovascular disease, hypertension and diabetes, and aerobic exercise*<br>*Excluded from the joint analysis.	5

ACLS, Aerobics Center Longitudinal Study; BMI, body mass index; CHAMP, Concord Health and Aging in Men Project; HABITAT, how areas in Brisbane Influence health and activity; MVPA, moderate-to-vigorous physical activity; NCEP-ATPIII, National Cholesterol Education Program- the third revision of the Adult Treatment Panel III; NDI, national death index; NHIS, National Health Interview Survey; NHS, Nurses’ Health Study; NHSII, Nurses’ Health Study II; NIH-AARP DHS, National Institutes of Health-American Association for Retired Persons Diet and Health Study

Appendix Table 5. List of publications excluded from meta-analysis because of multiple publication from the same cohort

Reference	Main reason for exclusion
**All-cause mortality**	
NHANES (**Porter et al. 2020[49] included**)	
Zhao et al. 2014[54]	Older publication year.
Loprinzi et al. 2015[55]	Older publication year.
Dankel et al. 2016 (a)[56]	Older publication year.
Dankel et al. 2016 (b)[57]	Older publication year.
Evenson et al. 2016[58]	Older publication year. Not adjusted for other physical activities.
NHIS (**Sheehan et al. 2020[18] included**)	
Schoenborn et al. 2011[53]	Older publication year.
Kraschnewski et al. 2016[59]	Older publication year.
**CVD**	
NHANES (**Porter et al. 2020[49] included**)	
Zhao et al. 2014[54]	Older publication year.
Loprinzi et al. 2015[55]	Older publication year.
Dankel et al. 2016 (a)[56]	Older publication year.
Evenson et al. 2016[58]	Older publication year. Not adjusted for other physical activities.
HPFS (**Grøntved et al. 2012[19] included**)	
Tanasescu et al. 2002[52]	Older publication year.

CVD, cardiovascular diseases; HPFS, Health Professionals Follow-Up Study; NHIS, National Health Interview Survey; NHANES, National Health and Nutrition Examination Survey

Appendix Table 6. Characteristics of the studies included in the meta-analysis

First author, year	Country; Cohort	Participants characteristics	Outcome; Case ascertainment; Case/Participants	Follow-up	Exposure measurement	Exposure category	Effect estimates	Covariates	Quality assessment
**All-cause mortality**									
Grøntved, 2012[19]	USA; HPFS	Men; 40-75 years	All-cause mortality; NDI, next of kin, or postal authorities; 6251/32 002	18 years (max)	Questionnaire	0 min/week<br>1-59 min/week<br>60-149 min/week<br>≥150 min/week	1<br>0.88 (0.83 to 0.94)<br>1.04 (0.93 to 1.17)<br>1.11 (0.90 to 1.37) (personal communication)	Age, smoking, alcohol consumption, coffee intake, race, family history of diabetes, intake of total energy, trans fat, polyunsaturated fat to saturated fat ratio, cereal fiber, whole grain, glycemic load, aerobic exercise, other physical activity of at least moderate intensity, and television viewing	7
Kamada, 2017[42]	USA; WHS	Women; ≥45 years (mean 62.2 years)	All-cause mortality; Family members, postal authorities medical records, death certificates, or NDI; 3055/28 879	12 years (mean)	Questionnaire	0 min/week<br>1-19 min/week<br>20-59 min/week<br>60-149 min/week<br>≥150 min/week<br><br>Aerobic MVPA (<150 min/week) & No strength training<br>Aerobic MVPA (≥150 min/week) & No strength training<br>Aerobic MVPA (<150 min/week) & Any strength training<br>Aerobic MVPA (≥150 min/week) & Any strength training	1<br>0.73 (0.65 to 0.82)<br>0.71 (0.62 to 0.82)<br>0.81 (0.67 to 0.97)<br>1.10 (0.77 to 1.56)<br><br>1<br>0.71 (0.64 to 0.79)<br>0.74 (0.65 to 0.85)<br>0.54 (0.47 to 0.61)	Age, trial randomization, race, education, postmenopausal status, hormone use, smoking status, parental history of myocardial infarction or cancer, alcohol intake, energy intake, saturated fat intake, fiber intake, fruit and vegetable intake, physical examination for screening, time per week spent in aerobic MVPA*, BMI, incidence of hypertension, high cholesterol, cardiovascular diseases, diabetes mellitus, and cancer before and during follow-up. *Excluded from the joint analysis.	7

First author, year	Country; Cohort	Participants characteristics	Outcome; Case ascertainment; Case/Participants	Follow-up	Exposure measurement	Exposure category	Effect estimates	Covariates	Quality assessment
Stamatakis, 2018[20]	England and Scotland; HSE and SHS	Men and women; ≥30 years (mean 45.6 years)	All-cause mortality; National Health Service Central Register; 5763/72 459	9.2 years (mean)	Questionnaire	No<br>Yes<br><br>None<br><66.0 min/week (women) and <52.5 min/week (men)<br>≥66.0 min/week (women) and ≥52.5 min/week (men)<br><br>Neither guideline<br>Aerobic only<br>Strength only<br>Both guideline	1<br>0.77 (0.69 to 0.87)<br><br>1<br>0.81 (0.69 to 0.95)<br>0.75 (0.64 to 0.88)<br><br>1<br>0.84 (0.78 to 0.90)<br>0.79 (0.66 to 0.94)<br>0.71 (0.57 to 0.87)	Age, sex, long-standing illness, alcohol consumption, psychological distress, BMI, smoking status, educational level, and weekly volume of other physical activity*<br>*Total volume of physical activity was alternatively included in the joint analysis	6
Liu, 2019[44]	USA; ACLS	Men and women; 18-89 years (mean 47 years)	All-cause mortality; NDI; 276/12 591	10.5 years (mean)	Questionnaire	0 min/week<br>1-59 min/week<br>60-119 min/week<br>≥120 min/week<br><br>0 time/week<br>1 time/week<br>2 times/week<br>3 times/week<br>≥4 times/week	1<br>0.64 (0.47 to 0.88)<br>0.84 (0.53 to 1.34)<br>1.03 (0.59 to 1.80)<br><br>1<br>0.65 (0.44 to 0.97)<br>0.68 (0.46 to 1.01)<br>0.67 (0.40 to 1.11)<br>1.29 (0.75 to 2.20)	Baseline examination year, age, sex, smoking status, alcohol consumption, parental history of CVD, BMI, aerobic exercise, hypertension, diabetes, and hypercholesterolemia	6
Sheehan, 2020[18]	USA; NHIS	Men and women; 18-84 years (mean 43.1 years)	All-cause mortality; National vital death registry (NHIS-LMF); 4955/26 727	17 years (max)	Interview	No<br>Yes	1<br>0.95 (0.85 to 1.07)	Age, sex, nativity status, census region of residence, marital status, race/ethnicity, educational attainment, household income, home ownership, smoking, drinking alcohol, BMI, self-reported health status, physical handicap, health condition, and other exercise types	7
Porter, 2020[49]	USA; NHANES	Men and women; ≥20 years (mean 46.3 years)	All-cause mortality; NDI; 3799/17 938	11.9 years (median)	Interview	No<br>Yes<br><br>0 min/week<br>1-59 min/week<br>≥60 min/week	1<br>0.89 (0.67 to 1.17)<br><br>1<br>0.75 (0.49 to 1.16)<br>0.98 (0.68 to 1.40)	Other leisure-time activities, age, gender, race, education, cigarette use, heavy alcohol consumption, BMI, household activity, transportation activity, and history of diabetes, arthritis, cancer, disability, and CVD	7

First author, year	Country; Cohort	Participants characteristics	Outcome; Case ascertainment; Case/Participants	Follow-up	Exposure measurement	Exposure category	Effect estimates	Covariates	Quality assessment
Patel, 2020[48]	USA; CPS-IINC	Men and women; 59-83 years (mean 70.2 years)	All-cause mortality; NDI; 18 034/72 462	13 years (max)	Questionnaire	0 min/week<br>1-59 min/week<br>60-119 min/week<br>≥120 min/week	1<br>0.88 (0.82 to 0.94)<br>0.90 (0.84 to 0.97)<br>1.01 (0.93 to 1.09)	Sex, age, BMI, survey type, education, self-reported overall health, smoking duration and intensity, alcohol use, marital status, work status, TV sitting time, aspirin use, and comorbidity score (reported personal history of high blood pressure, type 2 diabetes, and high cholesterol), and MVPA	6
Zhao, 2020[51]	USA; NHIS	Men and women; ≥18 years	All-cause mortality; NDI; 59 819/479 856	8.75 years (median)	Interview	Neither guideline<br>Aerobic only<br>Strength only<br>Both guideline	1<br>0.71 (0.69 to 0.72)<br>0.89 (0.85 to 0.94)<br>0.60 (0.57 to 0.62)	Sex, age, race/ethnicity, education, marital status, BMI, smoking status, alcohol intake, and chronic conditions (hypertension, heart disease, stroke, cancer, and diabetes)	5
**CVD**									
Grøntved, 2012[19]	USA; HPFS	Men; 40-75 years	CVD mortality; NDI, next of kin, or postal authorities; 1901/32 002	18 years (max)	Questionnaire	0 min/week<br>1-59 min/week<br>60-149 min/week<br>≥150 min/week	1<br>0.90 (0.80 to 1.01)<br>1.00 (0.80 to 1.26)<br>0.98 (0.63 to 1.51) (personal communication)	Age, smoking, alcohol consumption, coffee intake, race, family history of diabetes, intake of total energy, trans fat, polyunsaturated fat to saturated fat ratio, cereal fiber, whole grain, glycemic load, aerobic exercise, other physical activity of at least moderate intensity, and television viewing	7

First author, year	Country; Cohort	Participants characteristics	Outcome; Case ascertainment; Case/Participants	Follow-up	Exposure measurement	Exposure category	Effect estimates	Covariates	Quality assessment
Kamada, 2017[42]	USA; WHS	Women; ≥45 years (mean 62.2 years)	CVD mortality; Family members, postal authorities medical records, death certificates, or NDI; 411/28 879	12 years (mean)	Questionnaire	Aerobic MVPA (<150 min/week) & No strength training<br>Aerobic MVPA (≥150 min/week) & No strength training<br>Aerobic MVPA (<150 min/week) & Any strength training<br>Aerobic MVPA (≥150 min/week) & Any strength training	1<br>0.74 (0.56 to 0.98)<br>0.75 (0.53 to 1.07)<br>0.43 (0.29 to 0.63)	Age, trial randomization, race, education, postmenopausal status, hormone use, smoking status, parental history of myocardial infarction or cancer, alcohol intake, energy intake, saturated fat intake, fiber intake, fruit and vegetable intake, physical examination for screening, BMI, incidence of hypertension, high cholesterol, cardiovascular diseases, diabetes mellitus, and cancer before and during follow-up	7
Shiroma, 2017[43]	USA, WHS	Women; 47-97.8 years (mean 62.6 years)	CVD incidence or mortality; Annual follow-up questionnaires and medical records; 1742/35 754	10.7 years (mean)	Questionnaire	0 min/week<br>1-19 min/week<br>20-59 min/week<br>60-119 min/week<br>≥120 min/week	1<br>0.82 (0.64 to 1.06)<br>0.94 (0.73 to 1.21)<br>0.76 (0.59 to 0.98)<br>0.97 (0.70 to 1.33)	Age, smoking status, dietary habits, alcohol intake, postmenopausal status, hormone use, parental history of myocardial infarction, trial randomization, time per week spent in lower-intensity activities and aerobic activities, and BMI	6
Stamatakis, 2018[20]	England and Scotland; HSE and SHS	Men and women; ≥30 years (mean 45.6 years)	CVD mortality; National Health Service Central Register; 1723/73 937	9.2 years (mean)	Questionnaire	No<br>Yes<br><br>None<br><66.0 min/week (women) and <52.5 min/week (men)<br>≥66.0 min/week (women) and ≥52.5 min/week (men)<br><br>Neither guideline<br>Aerobic only<br>Strength only<br>Both guideline	1<br>0.88 (0.71 to 1.08)<br><br>1<br>0.89 (0.67 to 1.19)<br>0.86 (0.65 to 1.14)<br><br>1<br>0.78 (0.68 to 0.90)<br>0.89 (0.65 to 1.14)<br>0.77 (0.53 to 1.14) (personal communication)	Age, sex, long-standing illness, alcohol consumption, psychological distress, BMI, smoking status, educational level, and weekly volume of other physical activity<br>*Total volume of physical activity was alternatively included in the joint analysis	6

First author, year	Country; Cohort	Participants characteristics	Outcome; Case ascertainment; Case/Participants	Follow-up	Exposure measurement	Exposure category	Effect estimates	Covariates	Quality assessment
Liu, 2019[44]	USA; ACLS	Men and women; 18-89 years (mean 47 years)	CVD mortality or CVD morbidity; NDI for CVD mortality and mail-back health surveys for CVD morbidity; 127/12 591	10.5 years (mean)	Questionnaire	0 min/week<br>1-59 min/week<br>60-119 min/week<br>≥120 min/week<br><br>0 time/week<br>1 time/week<br>2 times/week<br>3 times/week<br>≥4 times/week	1<br>0.35 (0.24 to 0.51)<br>0.63 (0.39 to 1.03)<br>0.93 (0.51 to 1.68)<br><br>1<br>0.28 (0.17 to 0.46)<br>0.46 (0.29 to 0.70)<br>0.57 (0.33 to 0.96)<br>1.33 (0.75 to 2.36)	Baseline examination year, age, sex, smoking status, alcohol consumption, parental history of CVD, BMI, aerobic exercise, hypertension, diabetes, and hypercholesterolemia	6
Porter, 2019[46]	USA, ARICS	Men and women; 45-64 years (mean 54 years)	CVD incidence or mortality; Annual interviews, study visits, and community-wide surveillance of hospitalization discharge listings; 3966/13 204	25.2 years (median)	Interviewer-administered questionnaire	No<br>Yes	1<br>0.81 (0.62 to 1.02)	Marital status, income, race by study site, smoking, alcohol, education, age*sex, TV watching, BMI, active transportation, and total sport/exercise minutes/week minus minutes/week for weight training	7
Porter, 2020[49]	USA; NHANES	Men and women; ≥20 years (mean 46.3 years)	CVD mortality; NDI; 827/17 938	11.9 years (median)	Interview	No<br>Yes	1<br>0.53 (0.21 to 1.29)	Other leisure-time activities, age, gender, race, education, cigarette use, heavy alcohol consumption, BMI, household activity, transportation activity, and history of diabetes, arthritis, cancer, disability, and CVD	7
Patel, 2020[48]	USA; CPS-IINC	Men and women; 59-83 years (mean 70.2 years)	CVD mortality; NDI; 5770/72 462	13 years (max)	Questionnaire	0 min/week<br>1-59 min/week<br>60-119 min/week<br>≥120 min/week	1<br>0.81 (0.71 to 0.92)<br>0.98 (0.86 to 1.10)<br>1.03 (0.90 to 1.19)	Sex, age, BMI, survey type, education, self-reported overall health, smoking duration and intensity, alcohol use, marital status, work status, TV sitting time, aspirin use, and comorbidity score (reported personal history of high blood pressure, type 2 diabetes, and high cholesterol), and MVPA	6

First author, year	Country; Cohort	Participants characteristics	Outcome; Case ascertainment; Case/Participants	Follow-up	Exposure measurement	Exposure category	Effect estimates	Covariates	Quality assessment
Zhao, 2020[51]	USA; NHIS	Men and women; ≥18 years	CVD mortality; NDI; 13 509/479 856	8.75 years (median)	Interview	Neither guideline<br>Aerobic only<br>Strength only<br>Both guideline	1<br>0.65 (0.62 to 0.69)<br>0.82 (0.74 to 0.92)<br>0.50 (0.46 to 0.56)	Sex, age, race/ethnicity, education, marital status, BMI, smoking status, alcohol intake, and chronic conditions (hypertension, heart disease, stroke, cancer, and diabetes)	5
**Total cancer**									
Kamada, 2017[42]	USA; WHS	Women; ≥45 years (mean 62.2 years)	Cancer mortality; Family members, postal authorities medical records, death certificates, or NDI; 748/28 879	12 years (mean)	Questionnaire	0 min/week<br>1-59 min/week<br>≥60 min/week<br><br>Aerobic MVPA (<150 min/week) & No strength training<br>Aerobic MVPA (≥150 min/week) & No strength training<br>Aerobic MVPA (<150 min/week) & Any strength training<br>Aerobic MVPA (≥150 min/week) & Any strength training	1<br>0.87 (0.73 to 1.05)<br>0.92 (0.68 to 1.24)<br><br>1<br>0.97 (0.79 to 1.19)<br>0.91 (0.70 to 1.18)<br>0.93 (0.74 to 1.17)	Age, trial randomization, race, education, postmenopausal status, hormone use, smoking status, parental history of myocardial infarction or cancer, alcohol intake, energy intake, saturated fat intake, fiber intake, fruit and vegetable intake, physical examination for screening, time per week spent in aerobic MVPA* BMI, incidence of hypertension, high cholesterol, cardiovascular diseases, diabetes mellitus, and cancer before and during follow-up.<br>*Excluded from the joint analysis.	7
Stamatakis, 2018[20]	England and Scotland; HSE and SHS	Men and women; ≥30 years (mean 45.6 years)	Cancer mortality; National Health Service Central Register; 2089/77 195	9.2 years (mean)	Questionnaire	No<br>Yes<br><br>None<br><66.0 min/week (women) and <52.5 min/week (men)<br>≥66.0 min/week (women) and ≥52.5 min/week (men)<br><br>Neither guideline<br>Aerobic only<br>Strength only<br>Both guideline	1<br>0.69 (0.57 to 0.84)<br><br>1<br>0.72 (0.58 to 0.93)<br>0.67 (0.52 to 0.88)<br><br>1<br>0.99 (0.88 to 1.11)<br>0.66 (0.48 to 0.92)<br>0.70 (0.50 to 0.98) (personal communication)	Age, sex, long-standing illness, alcohol consumption, psychological distress, BMI, smoking status, educational level, and weekly volume of other physical activity<br>*Total volume of physical activity was alternatively included in the joint analysis	6

First author, year	Country; Cohort	Participants characteristics	Outcome; Case ascertainment; Case/Participants	Follow-up	Exposure measurement	Exposure category	Effect estimates	Covariates	Quality assessment
Siahpush, 2019[47]	USA; NHIS	Men and women; ≥18 years (mean 43.5 years)	Cancer mortality; NDI; 7275/310 282	7.9 years (mean)	Interview	0 time/week<br>1 time/week<br>2 times/week<br>3 times/week<br>4 times/week<br>≥5 times/week	1<br>0.89 (0.70 to 1.12)<br>0.77 (0.63 to 0.95)<br>0.82 (0.70 to 0.95)<br>0.71 (0.53 to 0.95)<br>0.84 (0.73 to 0.96)	Minutes of moderate physical activity, minutes of vigorous physical activity, smoking status, BMI, previous cancer diagnosis, chronic condition, self-rated health, sex, age, marital status, race/ethnicity, nativity, poverty status, and education	7
Porter, 2020[49]	USA; NHANES	Men and women; ≥20 years (mean 46.3 years)	Cancer mortality; NDI; 945/17 938	11.9 years (median)	Interview	No<br>Yes	1<br>0.81 (0.46 to 1.42)	Other leisure-time activities, age, gender, race, education, cigarette use, heavy alcohol consumption, BMI, household activity, transportation activity, and history of diabetes, arthritis, cancer, disability, and CVD	7
Patel, 2020[48]	USA; CPS-IINC	Men and women; 59-83 years (mean 70.2 years)	Cancer mortality; NDI; 5038/72 462	13 years (max)	Questionnaire	0 min/week<br>1-59 min/week<br>60-119 min/week<br>≥120 min/week	1<br>0.92 (0.81 to 1.04)<br>0.94 (0.83 to 1.06)<br>1.02 (0.89 to 1.17)	Sex, age, BMI, survey type, education, self-reported overall health, smoking duration and intensity, alcohol use, marital status, work status, TV sitting time, aspirin use, and comorbidity score (reported personal history of high blood pressure, type 2 diabetes, and high cholesterol), and MVPA	6
Rezende, 2020[50]	USA, HPSF	Men; 40-75 years (mean 67.5 years)	Total cancer incidence; Self-reported cancer diagnosis confirmed from medical records or NDI; 5158/33 787	24 years (max)	Questionnaire	None<br>Any<br><br>None<br>1-59 min/week<br>≥60 min/week<br><br>Per 60 min/week increase	1<br>0.98 (0.92 to 1.05)<br><br>1<br>0.98 (0.91 to 1.05)<br>0.99 (0.90 to 1.10)<br><br>1.01 (0.97 to 1.05)	Age, race, height, family history of cancer, physical exam in past two years, history of colonoscopy or sigmoidoscopy, smoking in pack years, regular aspirin use, multivitamin use, alcohol consumption, red and processed meat intake, Alternate Healthy Eating Index, prostate-specific antigen test in past 2 years, total physical activity except for resistance training, total energy intake, and BMI	7

First author, year	Country; Cohort	Participants characteristics	Outcome; Case ascertainment; Case/Participants	Follow-up	Exposure measurement	Exposure category	Effect estimates	Covariates	Quality assessment
Zhao, 2020[51]	USA; NHIS	Men and women; ≥18 years	Cancer mortality; NDI; 14 375/479 856	8.75 years (median)	Interview	Neither guideline<br>Aerobic only<br>Strength only<br>Both guideline	1<br>0.76 (0.73 to 0.80)<br>0.85 (0.77 to 0.95)<br>0.60 (0.56 to 0.65)	Sex, age, race/ethnicity, education, marital status, BMI, smoking status, alcohol intake, and chronic conditions (hypertension, heart disease, stroke, cancer, and diabetes)	5
**Diabetes**									
Grøntved, 2012[19]	USA; HPFS	Men; 40-75 years	Type 2 diabetes incidence; Self-reported diagnosis; 2278/32 002	18 years (max)	Questionnaire	0 min/week<br>1-59 min/week<br>60-149 min/week<br>≥150 min/week<br><br>Per 60 min/week increase	1<br>0.92 (0.82 to 1.02)<br>0.82 (0.67 to 1.00)<br>0.71 (0.49 to 1.00)<br><br>0.87 (0.81 to 0.94)	Age, smoking, alcohol consumption, coffee intake, race, family history of diabetes, intake of total energy, trans fat, polyunsaturated fat to saturated fat ratio, cereal fiber, whole grain, glycemic load, aerobic exercise, other physical activity of at least moderate intensity, television viewing, and BMI	6
Grøntved, 2014[40]	USA; NHS&NHSII	Women; 36-81 years	Type 2 diabetes incidence; Self-reported diagnosis; 3491/99 316	8 years (max)	Questionnaire	None<br>1-29 min/week<br>30-59 min/week<br>60-150 min/week<br>>150 min/week<br><br>Per 60 min/week increase	1<br>0.83 (0.73 to 0.94)<br>0.96 (0.82 to 1.11)<br>0.82 (0.70 to 0.95)<br>0.74 (0.54 to 1.01)<br><br>0.95 (0.90 to 1.00)	Age, smoking, alcohol consumption, coffee intake, race, family history of diabetes, postmenopausal hormone use, intake of total energy, trans fat, polyunsaturated fat to saturated fat ratio, cereal fiber, wholegrain, glycemic load, oral contraceptive use, menopausal status, aerobic physical activity, lower intensity muscular conditioning exercises, and BMI	6
Kuwahara, 2015[41]	Japan; J-ECOH	Men and women; 30-64 years (mean 45.3 years)	Type 2 diabetes incidence; HbA1c ≥6.5%, fasting glucose ≥126 mg/dL, random plasma glucose ≥200 mg/dL, history of diabetes or current medication for diabetes; 1770/26 630	5.2 years (mean)	Questionnaire	No<br>Yes	1<br>0.70 (0.51 to 0.96)	Age, sex, smoking status, alcohol consumption, sleep duration, aerobic exercise, hypertension, shift work, occupational physical activity, family history of diabetes, and BMI	6

First author, year	Country; Cohort	Participants characteristics	Outcome; Case ascertainment; Case/Participants	Follow-up	Exposure measurement	Exposure category	Effect estimates	Covariates	Quality assessment
Shiroma, 2017[43]	USA, WHS	Women; 47-97.8 years (mean 62.6 years)	Type 2 diabetes incidence; Annual follow-up questionnaires confirmed from a telephone interview, supplemental questionnaire, and medical records; 2120/35 754	10.7 years (mean)	Questionnaire	0 min/week<br>1-19 min/week<br>20-59 min/week<br>60-119 min/week<br>≥120 min/week	1<br>0.74 (0.59 to 0.93)<br>0.91 (0.72 to 1.14)<br>0.76 (0.60 to 0.95)<br>0.76 (0.54 to 1.05)	Age, smoking status, dietary habits, alcohol intake, postmenopausal status, hormone use, parental history of myocardial infarction, trial randomization, time per week spent in lower-intensity activities and aerobic activities, and BMI	6
Mielke, 2020[17]	Australia; HABITAT	Men and women; 40-65 years	Type 2 diabetes incidence; Self-reported diabetes diagnosis; 267/8784	6 years (max)	Questionnaire	None<br><1 time/week<br>≥1 time/week	1<br>0.55 (0.32 to 0.93)<br>0.69 (0.45 to 1.05)<br>(personal communication)	Sex, age, education, annual income, living arrangements, cigarette smoking status, physical activity, hypertension, and obesity	4
**Colon cancer**									
Mazzilli, 2019[45]	USA, NIH-AARP DHS	Men and women; 50-71 years	Colon cancer incidence; Cancer registries; 1715 /215 122	10 years (max)	Questionnaire	None<br>Any<br><br>None<br>5-90 min/week<br>≥120 min/week<br><br>Low activity & No weight lifting<br>High activity & No weight lifting<br>Low activity & Any weight lifting<br>High activity & Any weight lifting<br>(Low activity: <7.5 MET-h/week)<br>(High activity: ≥7.5 MET-h/week)	1<br>0.95 (0.90 to 1.00)<br><br>1<br>0.75 (0.66 to 0.87)<br>0.78 (0.61 to 0.98)<br><br>1<br>0.93 (0.83 to 1.03)<br>0.77 (0.57 to 1.03)<br>0.69 (0.60 to 0.80)	Age, sex, BMI, smoking status, race, education, alcohol intake, and MVPA not including weight lifting*<br>*Excluded from the joint analysis.	6

First author, year	Country; Cohort	Participants characteristics	Outcome; Case ascertainment; Case/Participants	Follow-up	Exposure measurement	Exposure category	Effect estimates	Covariates	Quality assessment
Rezende, 2020[50]	USA, HPSF	Men; 40-75 years (mean 67.5 years)	Colon cancer incidence; Self-reported cancer diagnosis confirmed from medical records or NDI; 700/33 787	24 years (max)	Questionnaire	None<br>Any<br><br>None<br>1-59 min/week<br>≥60 min/week<br><br>Per 60 min/week increase<br><br>Low activity & No resistance training<br>High activity & No resistance training<br>Low activity & Any resistance training<br>High activity & Any resistance training<br>(Low activity: <16 MET-h/week)<br>(High activity: ≥16 MET-h/week)	1<br>1.04 (0.87 to 1.25)<br><br>1<br>0.94 (0.77 to 1.16)<br>1.32 (1.01 to 1.72)<br><br>1.12 (1.02 to 1.22)<br><br>1<br>0.88 (0.73 to 1.06)<br>0.84 (0.57 to 1.25)<br>0.95 (0.77 to 1.19)	Age, race, height, family history of cancer, physical exam in past two years, history of colonoscopy or sigmoidoscopy, smoking in pack years, regular aspirin use, multivitamin use, alcohol consumption, red and processed meat intake, Alternate Healthy Eating Index, prostate-specific antigen test in past 2 years, total physical activity except for resistance training*, total energy intake, and BMI<br>*Excluded from the joint analysis.	7
**Kidney cancer**									
Mazzilli, 2019[45]	USA, NIH-AARP DHS	Men and women; 50-71 years	Kidney cancer incidence; Cancer registries; 851/215 122	10 years (max)	Questionnaire	None<br>5-90 min/week<br>≥120 min/week	1<br>0.94 (0.78 to 1.12)<br>0.80 (0.59 to 1.11)	Age, sex, BMI, smoking status, race, education, alcohol intake, and MVPA not including weight lifting	6
Rezende, 2020[50]	USA, HPSF	Men; 40-75 years (mean 67.5 years)	Kidney cancer incidence; Self-reported cancer diagnosis confirmed from medical records or NDI; 212/33 787	24 years (max)	Questionnaire	None<br>Any<br><br>None<br>1-59 min/week<br>≥60 min/week<br><br>Per 60 min/week increase	1<br>0.80 (0.58 to 1.11)<br><br>1<br>0.89 (0.63 to 1.26)<br>0.58 (0.32 to 1.04)<br><br>0.78 (0.58 to 1.04)	Age, race, height, family history of cancer, physical exam in past two years, history of colonoscopy or sigmoidoscopy, smoking in pack years, regular aspirin use, multivitamin use, alcohol consumption, red and processed meat intake, Alternate Healthy Eating Index, prostate-specific antigen test in past 2 years, total physical activity except for resistance training, total energy intake, and BMI	7

First author, year	Country; Cohort	Participants characteristics	Outcome; Case ascertainment; Case/Participants	Follow-up	Exposure measurement	Exposure category	Effect estimates	Covariates	Quality assessment
**Bladder cancer**									
Mazzilli, 2019[45]	USA, NIH-AARP DHS	Men and women; 50-71 years	Bladder cancer incidence; Cancer registries; 1836/215 122	10 years (max)	Questionnaire	None<br>5-90 min/week<br>≥120 min/week	1<br>0.97 (0.86 to 1.10)<br>0.98 (0.81 to 1.19)	Age, sex, BMI, smoking status, race, education, alcohol intake, and MVPA not including weight lifting	6
Rezende, 2020[50]	USA, HPSF	Men; 40-75 years (mean 67.5 years)	Bladder cancer incidence; Self-reported cancer diagnosis confirmed from medical records or NDI; 505/33 787	24 years (max)	Questionnaire	None<br>Any<br><br>None<br>1-59 min/week<br>≥60 min/week<br><br>Per 60 min/week increase	1<br>0.85 (0.69 to 1.05)<br><br>1<br>0.94 (0.75 to 1.18)<br>0.61 (0.42 to 0.90)<br><br>0.80 (0.66 to 0.96)	Age, race, height, family history of cancer, physical exam in past two years, history of colonoscopy or sigmoidoscopy, smoking in pack years, regular aspirin use, multivitamin use, alcohol consumption, red and processed meat intake, Alternate Healthy Eating Index, prostate-specific antigen test in past 2 years, total physical activity except for resistance training, total energy intake, and BMI	7
**Lung cancer**									
Mazzilli, 2019[45]	USA, NIH-AARP DHS	Men and women; 50-71 years	Lung cancer incidence; Cancer registries; 3480/215 122	10 years (max)	Questionnaire	None<br>5-90 min/week<br>≥120 min/week	1<br>0.91 (0.82 to 1.00)<br>0.90 (0.81 to 1.12)	Age, sex, BMI, smoking status, race, education, alcohol intake, and MVPA not including weight lifting	6
Rezende, 2020[50]	USA, HPSF	Men; 40-75 years (mean 67.5 years)	Lung cancer incidence; Self-reported cancer diagnosis confirmed from medical records or NDI; 595/33 787	24 years (max)	Questionnaire	None<br>Any<br><br>None<br>1-59 min/week<br>≥60 min/week<br><br>Per 60 min/week increase	1<br>0.87 (0.71 to 1.07)<br><br>1<br>0.86 (0.69 to 1.09)<br>0.90 (0.63 to 1.27)<br><br>0.93 (0.79 to 1.09)	Age, race, height, family history of cancer, physical exam in past two years, history of colonoscopy or sigmoidoscopy, smoking in pack years, regular aspirin use, multivitamin use, alcohol consumption, red and processed meat intake, Alternate Healthy Eating Index, prostate-specific antigen test in past 2 years, total physical activity except for resistance training, total energy intake, and BMI	7

First author, year	Country; Cohort	Participants characteristics	Outcome; Case ascertainment; Case/Participants	Follow-up	Exposure measurement	Exposure category	Effect estimates	Covariates	Quality assessment
**Pancreas cancer**									
Mazzilli, 2019[45]	USA, NIH-AARP DHS	Men and women; 50-71 years	Pancreas cancer incidence; Cancer registries; 795/215 122	10 years (max)	Questionnaire	None<br>5-90 min/week<br>≥120 min/week	1<br>1.15 (0.96 to 1.37)<br>0.98 (0.71 to 1.34)	Age, sex, BMI, smoking status, race, education, alcohol intake, and MVPA not including weight lifting	6
Rezende, 2020[50]	USA, HPSF	Men; 40-75 years (mean 67.5 years)	Pancreas cancer incidence; Self-reported cancer diagnosis confirmed from medical records or NDI; 233/33 787	24 years (max)	Questionnaire	None<br>Any<br><br>None<br>1-59 min/week<br>≥60 min/week<br><br>Per 60 min/week increase	1<br>1.15 (0.85 to 1.56)<br><br>1<br>1.13 (0.81 to 1.57)<br>1.22 (0.76 to 1.96)<br><br>1.01 (0.84 to 1.23)	Age, race, height, family history of cancer, physical exam in past two years, history of colonoscopy or sigmoidoscopy, smoking in pack years, regular aspirin use, multivitamin use, alcohol consumption, red and processed meat intake, Alternate Healthy Eating Index, prostate-specific antigen test in past 2 years, total physical activity except for resistance training, total energy intake, and BMI	7

ACLS, Aerobics Center Longitudinal Study; ARICS, Atherosclerosis Risk in Communities Study; BMI, body mass index; CHD, coronary heart disease; CVD, cardiovascular disease; HABITAT, how areas in Brisbane Influence health and activity; HPFS, Health Professionals Follow-Up Study; HSE, Health Survey for England; J-ECOHS, Japan epidemiology collaboration on occupational health study; MI, myocardial infarction; MVPA, moderate-to-vigorous physical activity; NDI, national death index; NHIS, National Health Interview Survey; NHIS-LMF, National Health Interview Survey-Linked Mortality Files; NHANES, National Health and Nutrition Examination Survey; NHS, Nurses' Health Study; NHSII, Nurses' Health Study II; NIH-AARP DHS, National Institutes of Health-American Association for Retired Persons Diet and Health Study; SHS, Scottish Health Survey; TV, television; WHS, Women's Health Study

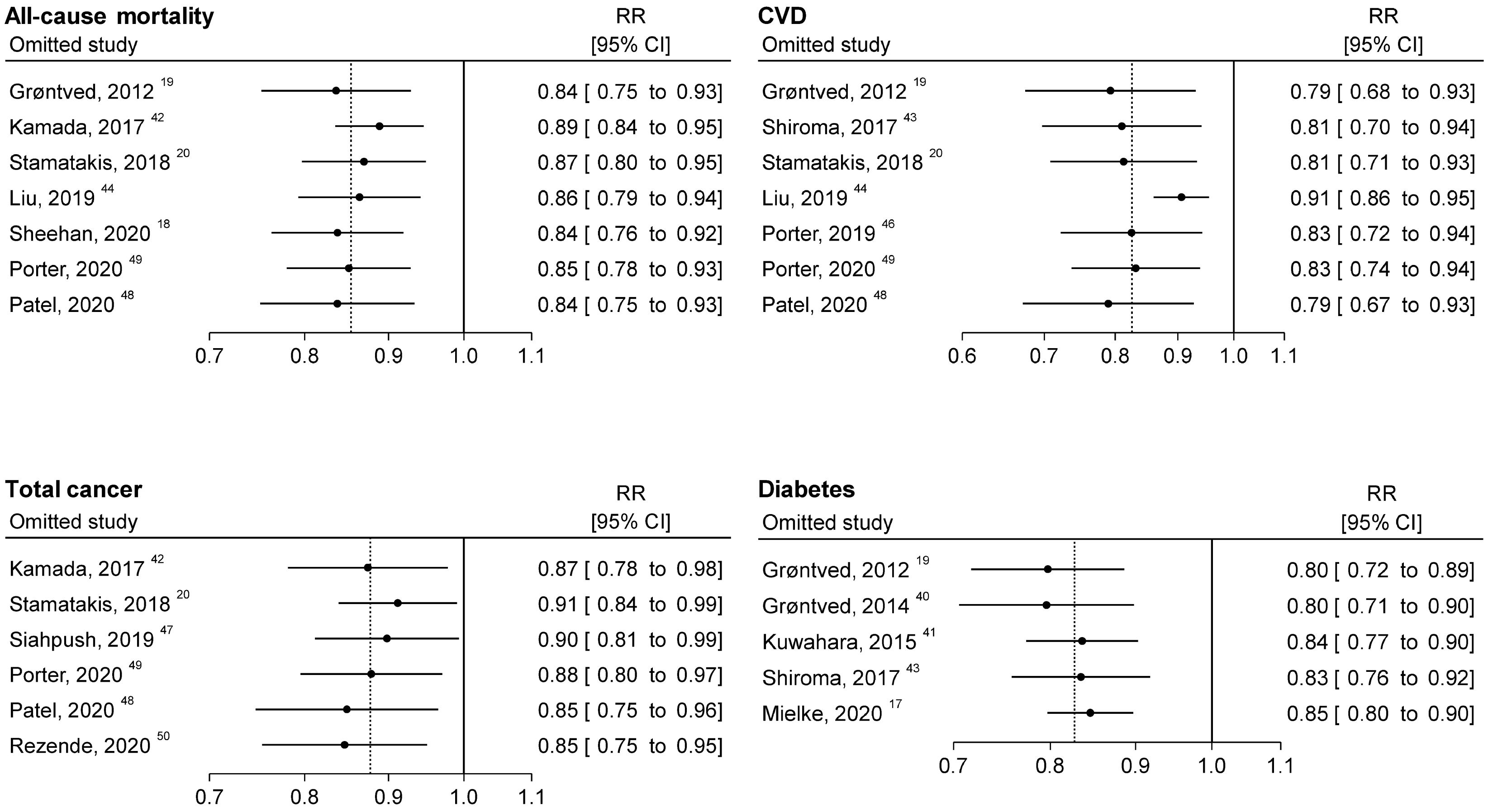

Appendix Figure 1. Leave-one-out analysis for the associations of muscle-strengthening activities (two-group analysis) with all-cause mortality, CVD, total cancer, and diabetes. For CVD, the heterogeneity disappeared (I^2=0.0%) when the study by Liu et al. was excluded (I^2=0.0%). For diabetes, when the study by Mielke et al.[17] with low quality (NOS=4) was excluded, the heterogeneity substantially reduced (I^2=9.5%). CI=confidence intervals; CVD=cardiovascular diseases; NOS=Newcastle-Ottawa Scale; RR=relative risk.

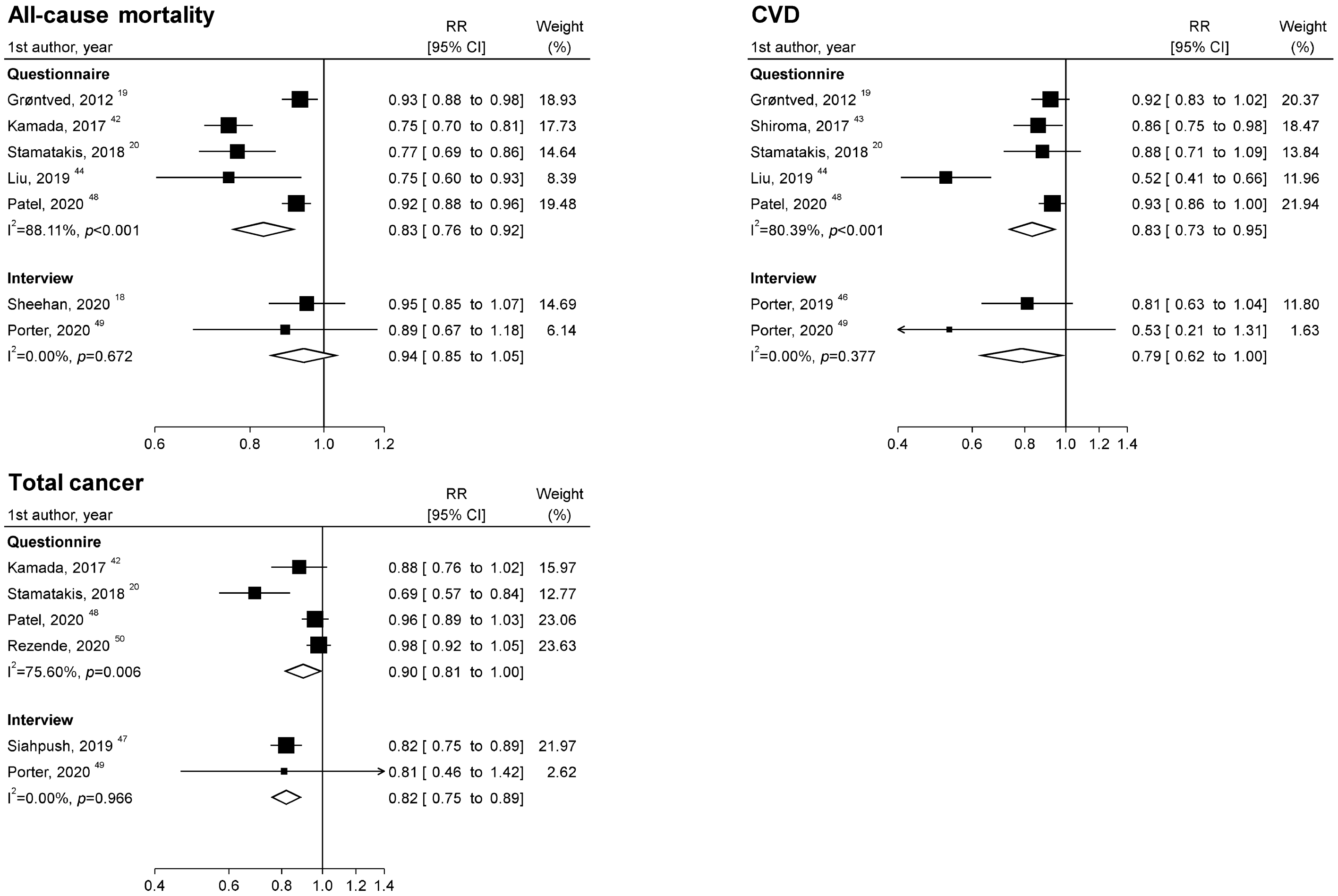

Appendix Figure 2. Forest plot of subgroup analysis by the exposure assessment (questionnaire or interview) for the association of muscle-strengthening activities (two-group analysis) with all-cause mortality, CVD, and total cancer. Diamonds indicate overall RRs with 95% CI. CI=confidence intervals; CVD=cardiovascular diseases; RR=relative risk.

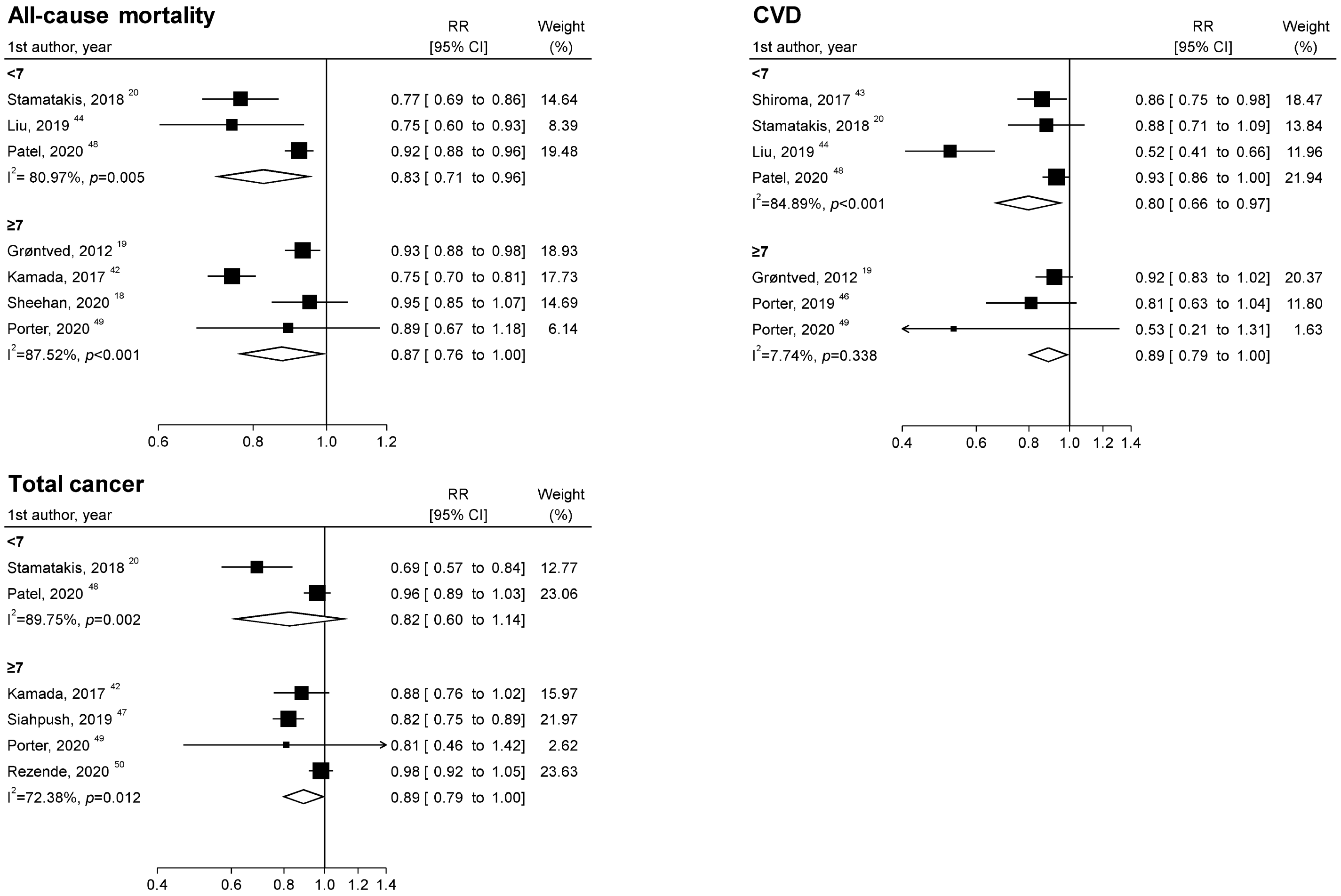

Appendix Figure 3. Forest plot of subgroup analysis by the quality score of Newcastle-Ottawa Scale (<7 or ≥7) for the association of muscle-strengthening activities (two-group analysis) with all-cause mortality, CVD, and total cancer. Diamonds indicate overall RRs with 95% CI. CI=confidence interval; CVD=cardiovascular diseases; RR=relative risk.

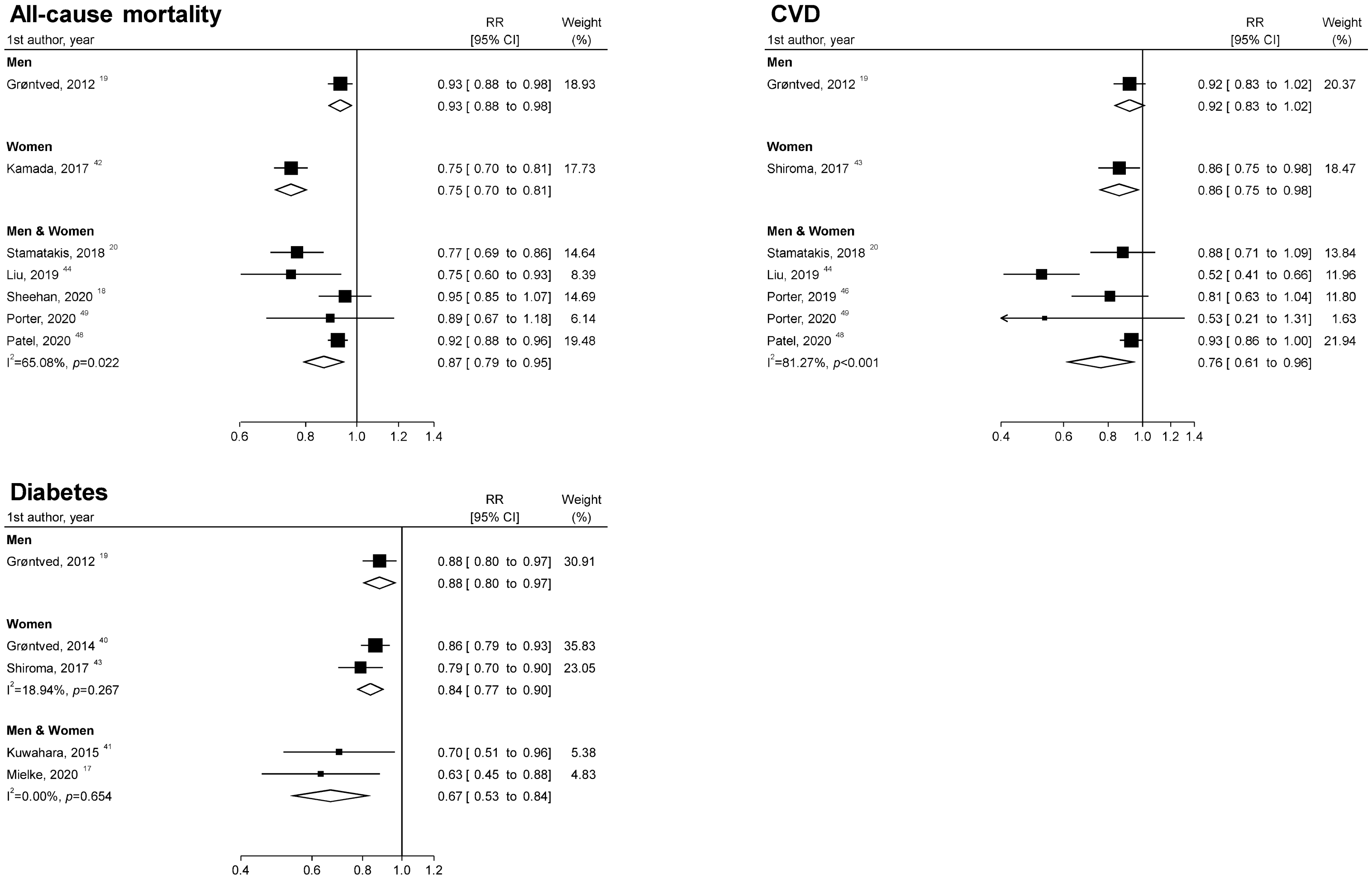

Appendix Figure 4. Forest plot of subgroup analysis by sex for the association of muscle-strengthening activities (two-group analysis) with all-cause mortality, CVD, and diabetes. Diamonds indicate overall RRs with 95% CI. CI=confidence interval; CVD=cardiovascular diseases; RR=relative risk

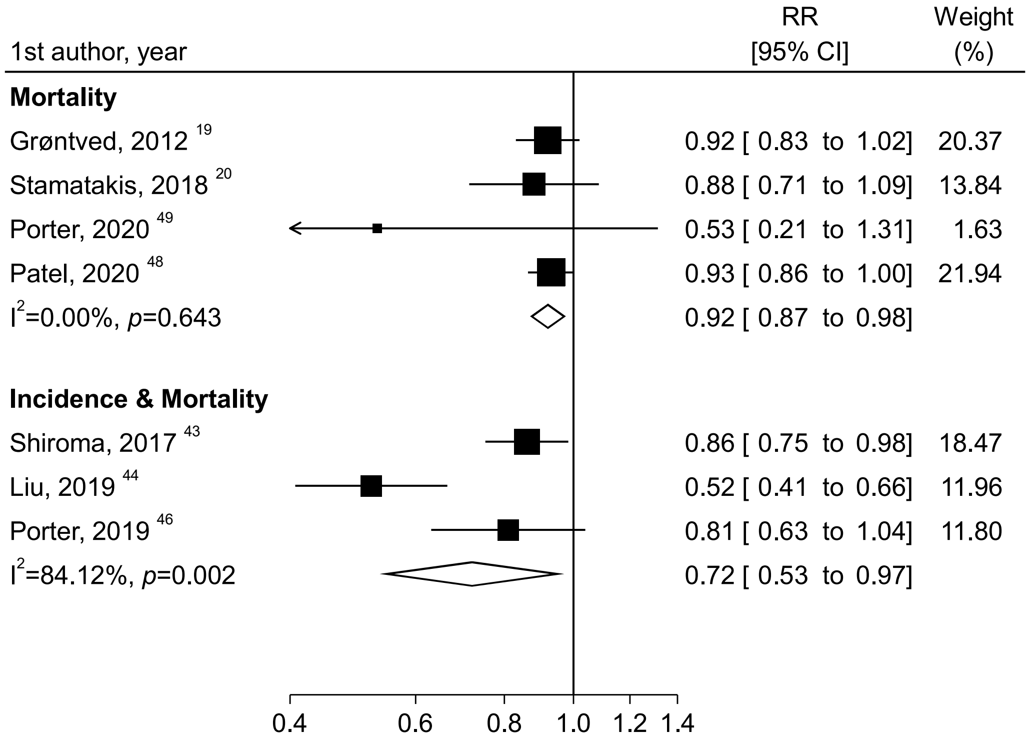

Appendix Figure 5. Forest plot of subgroup analysis by the type of case for the association of muscle-strengthening activities (two-group analysis) with CVD. Diamonds indicate overall RRs with 95% CI. CI=confidence interval; CVD=cardiovascular diseases; RR=relative risk.

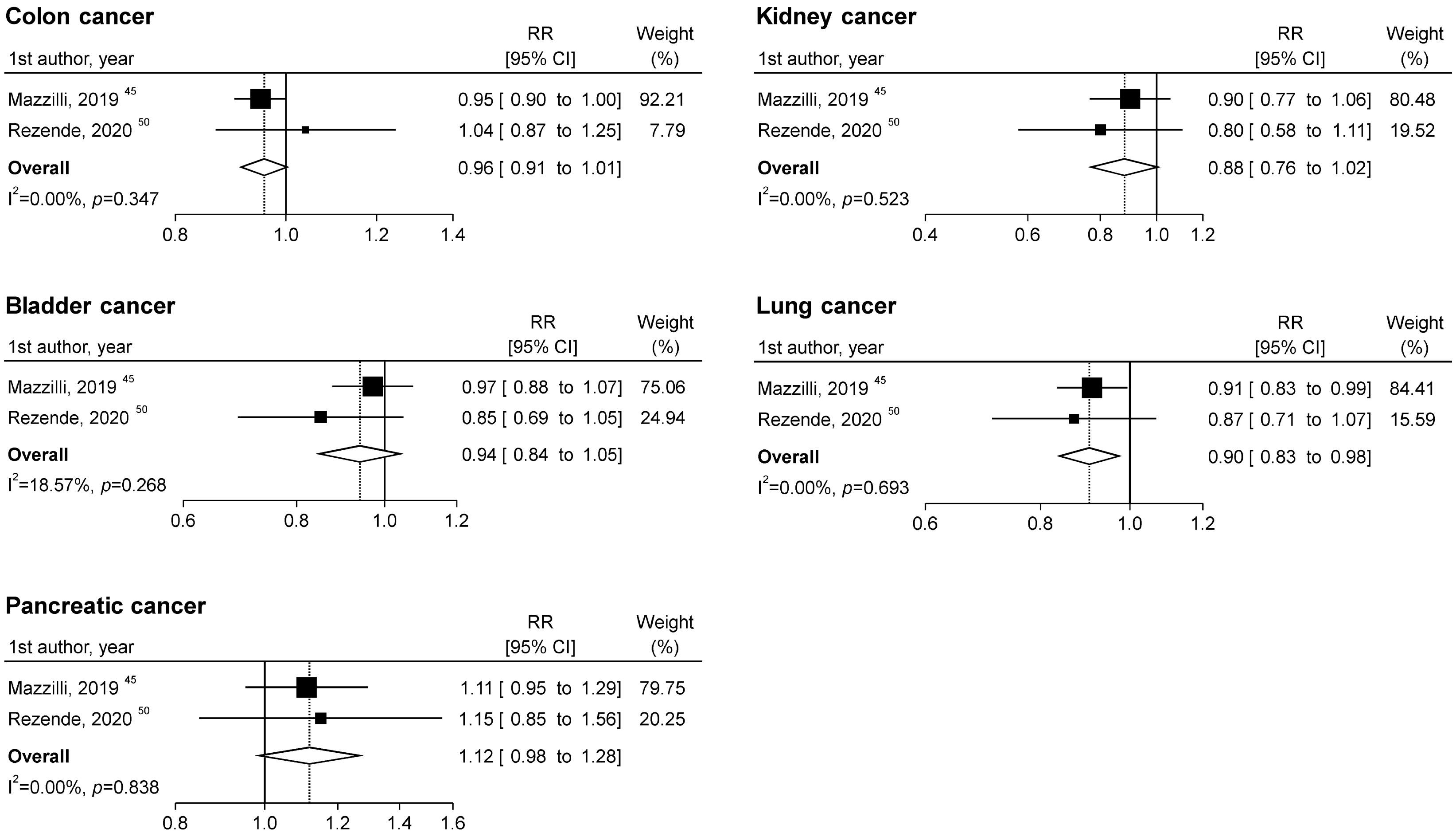

Appendix Figure 6. Forest plot for the associations of muscle-strengthening activities (two-group analysis) with site-specific cancers incidence. Diamonds indicate overall RRs with 95% CI. CI=confidence interval; RR=relative risk.

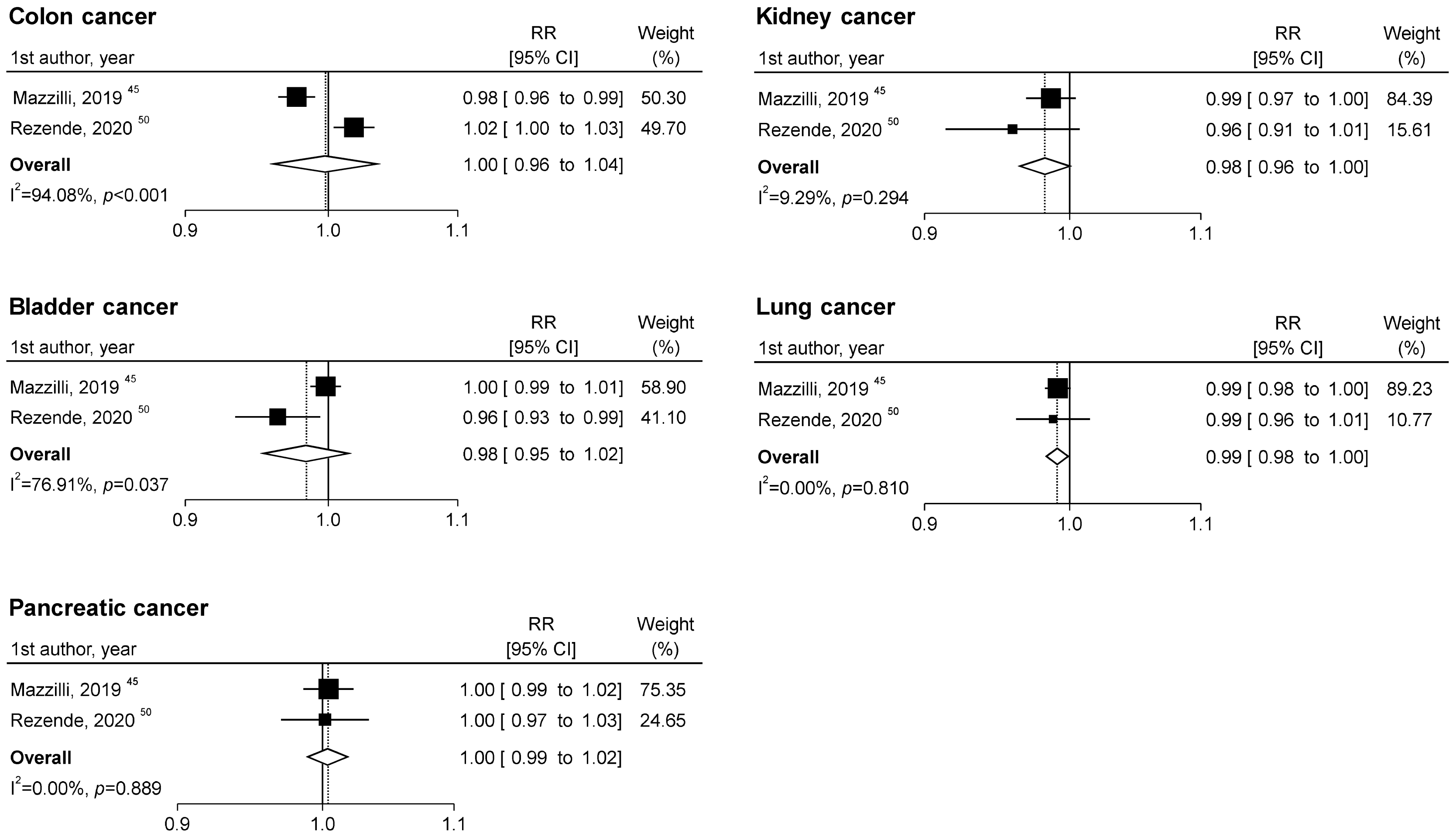

Appendix Figure 7. Forest plot for the linear dose-response association of muscle-strengthening activities (per 10-min/week increase) with site-specific cancers incidence. Diamonds indicate overall RRs with 95% CI. CI=confidence interval; RR=relative risk.
